# Effects of Different Storage Conditions on Lipid Stability in Mice Tissue Homogenates

**DOI:** 10.3390/metabo13040504

**Published:** 2023-03-31

**Authors:** Erika Dorochow, Robert Gurke, Samuel Rischke, Gerd Geisslinger, Lisa Hahnefeld

**Affiliations:** 1 Pharmazentrum Frankfurt/ZAFES, Institute of Clinical Pharmacology, Johann Wolfgang Goethe University, Theodor Stern-Kai 7, 60590 Frankfurt am Main, Germany; 2 Fraunhofer Institute for Translational Medicine and Pharmacology ITMP, and Fraunhofer Cluster of Excellence for Immune Mediated Diseases CIMD, Theodor-Stern-Kai 7, 60596 Frankfurt am Main, Germany

**Keywords:** lipids, stability, lipidomics, lipolytic ratios, tissue, homogenization, pre-analytics, liquid chromatography, mass spectrometry

## Abstract

Lipids are biomolecules involved in numerous (patho-)physiological processes and their elucidation in tissue samples is of particular interest. However, tissue analysis goes hand in hand with many challenges and the influence of pre-analytical factors can intensively change lipid concentrations ex vivo, compromising the results of the whole research project. Here, we study the influence of pre-analytical factors on lipid profiles during the processing of homogenized tissues. Homogenates from four different mice tissues (liver, kidney, heart, spleen) were stored at room temperature as well as in ice water for up to 120 min and analyzed via ultra-high-performance liquid chromatography-high-resolution mass spectrometry (UHPLC-HRMS). Lipid class ratios were calculated since their suitability as indicators for sample stability has been previously illustrated. Only approx. 40% of lipid class ratios were unchanged after 35 min, which was further reduced to 25% after 120 min during storage at room temperature. In contrast, lipids in tissue homogenates were generally stable when samples were kept in ice water, as more than 90% of investigated lipid class ratios remained unchanged after 35 min. Ultimately, swift processing of tissue homogenates under cooled conditions represents a viable option for lipid analysis and pre-analytical factors require more attention to achieve reliable results.

## 1. Introduction

Lipids are biomolecules that play an important role in many processes in the biological context, e.g., affecting inflammation in chronic diseases [1,2]. Whereas matrices such as plasma, which reflect the systemic lipid profile, are used for biomarker discovery studies, elucidation of the underlying pathophysiological processes and discovery of potential new drug targets often require a more focused look at the affected organ. Therefore, the latter investigations usually rely on animal experiments, analyzing the distinct lipid profiles of the organ tissue and their disease-specific alterations [3]. Yet, there are many challenges on the way to obtaining reliable lipidomics results from tissue samples. Starting with the method of euthanasia and the time needed for dissection, every step during sample preparation can impact the results [4,5]. One of the challenges in tissue lipidomics is the standardization of the sample amount. (Relative) quantitative lipidomics using LC-MS usually require only a small but comparable sample amount. Many tissues such as kidney or brain are structured, and dissection of comparable samples is difficult. Therefore, the tissue can be homogenized before sampling with several methods available. Cryogrinding and lyophilization are suitable options, but they require expensive lab equipment and, in the case of lyophilization, are also time-consuming [6]. Furthermore, weighing in exactly the same, very small amount of homogenized tissue is hardly possible and requires a well-calibrated analytical balance with low minimum weight requirements. A simpler and often employed approach is tissue homogenization via wet grinding, which allows the sampling of comparable tissue amounts by volume. Here, the choice of extraction solvent, the tissue concentration, and the homogenization process are important to properly extract lipids [7,8].

The ultimate goal of disease-related lipidomic analysis is the translation into clinical practice, a process that is only slowly progressing so far [9,10,11,12,13]. This is partly caused by pre-analytical factors that impair lipidomics results [4,14] and that have hardly been explored [15]. Tissue lipidomics is no exception, especially as tissue homogenization leads to the physical disruption of the matrix integrity and change in sample stability. Krautbauer and colleagues showed that the lipid profile in tissue homogenates created by wet grinding is greatly affected by storage at room temperature (RT) [16]. They proposed to improve lipid stability by the addition of sodium dodecyl sulfate (SDS) to tissue homogenates [16]—a procedure that has presumably not been introduced into common practice yet as it requires the presence of an additional agent that might interfere with the analysis.

One of the most commonly applied procedures to improve lipid stability is storage on ice [17]. Yet, so far, no data are available on how the lipid profile of tissue homogenates is affected by storage under cooled conditions. Hence, in this study, we investigate the time-dependent changes of lipid profiles in various murine tissue homogenates, i.e., liver, kidney, heart, and spleen homogenates that were stored at RT as well as in ice water (IW). Liver, kidney, heart, and spleen samples were chosen as they all represent tissue types with high metabolic activity [18,19,20,21] as well as (patho-)physiological relevance [3] and are also found in numerous studies [22,23,24,25]. We opted for 25% ethanol in water with the addition of 10 µM indometacin as extraction solvent since we found 25% ethanol a compromise between lipid solubility and avoiding protein precipitation. Furthermore, the addition of indometacin can efficiently stabilize homogenates by inhibiting enzymatic activity [4,26,27]. Tissue homogenates were stored for up to 120 min, reflecting standard processing times for tissue homogenates in lipidomics studies. The analysis of the samples was performed via ultra-high-performance liquid chromatography-high-resolution mass spectrometry (UHPLC-HRMS). The results were evaluated by applying the lipid class ratios previously described [16], as well as using additional ratios. These lipid class ratios were chosen because of a mechanistic linkage based on hydrolyzation of, e.g., phosphatidylcholines to lysophosphatidylcholines. This approach further increases the sensitivity of detecting alterations in the lipid profile.

## 2. Materials and Methods

### 2.1. Chemicals and Internal Standards

Acetonitrile (ACN), methanol (MeOH), methyl *tert*-butyl ether (MTBE), and water are LC-MS-grade solvents and were purchased from Carl Roth (Karlsruhe, Germany). Formic acid (98–100%) was acquired from AppliChem (Darmstadt, Germany). Ammonium formate (eluent additive for LC-MS, LiChropur™, ≥99.0%), ethanol (EtOH, for residue analysis, ≥99.8%), and indometacin (≥99%) were obtained from Sigma-Aldrich (St. Louis, MO, USA).

Internal standards solution included the following lipid standards: arachidonic acid-d8, CE 18:1-d7, Cer d18:1/16:0-d7, cholesterol-d7, DG 15:0/18:1-d7, LacCer d18:1/17:0, LPC 18:1-d7, LPC O-16:0-d4, LPE 18:1-d7, LPG 17:1, LPI 17:1, PC 15:0/18:1-d7, PC O-18:0/18:1-d9, PE 15:0/18:1-d7, PE O-18:0/18:1-d9, PG 15:0/18:1-d7, PI 15:0/18:1-d7, PS 15:0/18:1-d7, SM d18:1/18:1-d9, TG 14:0/16:1/14:0-d5, TG 15:0/18:1-d7/15:0, and TG 20:0/20:1/20:0-d5. Arachidonic acid-d8 and LPC O-16:0-d4 were purchased from Cayman Chemical (Ann Arbor, MI, USA). All other standards were obtained from Avanti Polar Lipids (Alabaster, AL, USA). For concentrations of used internal standards, please refer to the Supplementary Material (Data S1).

### 2.2. Animals and Sample Collection

Five female wild-type mice with a C57BL/6 genetic background were sacrificed to assess the lipid stability of tissue homogenates. The mice underwent cervical dislocation with a cardiac blood draw and organs (heart, spleen, liver, and kidney) were directly dissected and snap-frozen in liquid nitrogen. Tissue samples were stored at −80 °C until homogenization. The conduction of the experiments was approved by the local ethics committee for animal research (Darmstadt, Germany) and complied with the European and German regulations for animal research and the ARRIVE guidelines. Moreover, the “Principles of laboratory animal care” (NIH publication No. 86-23, revised 1985) were applied.

### 2.3. Tissue Homogenization

Wet bead milling with zirconium oxide beads (external diameter 2.8 mm) was employed for the homogenization of tissue samples. For this purpose, whole organs were transferred to reinforced 2 mL tubes (Bertin Technologies, Montigny-le-Bretonneux, France) containing 10 beads each. Based on the individual tissue weights, varying volumes of a pre-cooled extraction solvent (25% EtOH with 10 µM indometacin) were added to create initial homogenates with defined tissue concentrations (spleen: 0.3 mg/µL; liver and kidney: 0.15 mg/µL; heart: 0.1 mg/µL). Tubes were then placed into a Precellys 24-Dual homogenizer with a Cryolys cooling module (Bertin Technologies, Montigny-le-Bretonneux, France), which was cooled at <5 °C with dry ice. Tissue samples were homogenized at 6500× *g* for 20 s with two repetitions and 40 s pauses in between to avoid overheating. Initial homogenates were further diluted to tissue concentrations of 0.025 mg/µL using the same pre-cooled extraction solvent and a sample volume of 20 µL was used for lipid extraction.

### 2.4. Lipid Extraction

Lipids were extracted directly or after the specified incubation times at RT or in IW. An MTBE-based extraction method that was previously described by Matyash et al. was employed [28,29]. To 20 µL of diluted tissue homogenate (0.025 mg/µL), 75 µL of internal standards solved in MeOH, 250 µL of MTBE, and 50 µL of 50 mM ammonium formate were added. Samples were vortexed for 1 min and centrifuged at ambient temperature and 20,000× *g*. Afterwards, the layer containing organic solvent (upper layer) was transferred, whereas the layer containing water (lower layer) was re-extracted with 100 µL of MTBE:MeOH:water (10:3:2.5, *v*/*v*/*v*, upper layer), following subsequent centrifugation. Upper layers were combined and evaporated under a stream of nitrogen at 45 °C. Dried samples were stored at −80 °C and reconstituted with 100 µL of MeOH preceding analysis.

### 2.5. Lipid Profiling by UHPLC-HRMS

UHPLC-HRMS analysis was performed as previously described [30] using a Vanquish Horizon UHPLC system coupled to an Orbitrap Exploris 480 mass spectrometer (both Thermo Fisher Scientific, Dreieich, Germany). Study samples were analyzed in a randomized order and were chromatographically separated with a Zorbax RRHD Eclipse Plus C8 column (1.8 µm × 50 × 2.1 mm internal diameter, Agilent Technologies, Waldbronn, Germany) in combination with an equivalent pre-column by applying a 14 min binary gradient. Data were acquired using a heated electrospray ionization (H-ESI) source operated in positive and negative modes while scanning a range from 180 to 1500 *m*/*z* at 120,000 mass resolving power. MS^2^ spectra were collected via data-dependent acquisition at 15,000 mass resolving power with a total cycle time of 600 ms. The UHPLC-HRMS system was operated via XCalibur software v4.4 and tissue-specific lipids were identified in Compound Discoverer 3.1 (both Thermo Fisher Scientific, San Jose, CA, USA) using the LipidBlast VS68 positive and negative libraries. In TraceFinder software v5.1 (Thermo Fisher Scientific, San Jose, CA, USA), a relative quantitative method was set up based on the previously identified lipids and evaluation of the acquired data with a mass tolerance of 5 ppm was performed. For detailed information on the applied method, please refer to the Supplementary Material (Data S1).

### 2.6. Data Processing and Use of Quality Control (QC) Samples

For all measured analytes, areas were divided by the areas of corresponding internal standards, resulting in normalized data (area ratios) that were used for evaluation. In a tissue-wise manner, analytes with >20% missing values were excluded from evaluation. All remaining missing values were replaced with ½ of the minimum value of the corresponding analyte.

For kidney and heart homogenates, pooled quality control samples (*n* = 10) were employed to assess the quality of the analysis and to further filter the data. Similarly, for liver and spleen homogenates, relative changes between replicate measurements of t_0_ samples (*n* = 5) were utilized. Consequently, analytes showing >20% relative standard deviation (RSD) in quality control (QC) samples for kidney and heart homogenates and >15% relative change between two replicate measurements for liver and spleen homogenates were excluded from evaluation. In this manner, the number of evaluated lipids was different in investigated tissue types, but the same lipids were evaluated at every point in time for one specific tissue type.

In addition, a total of 50 replicate human plasma samples that were created from two individual pools were measured over the whole run time in an overlapping manner to monitor extraction reproducibility and system performance. A volume of 10 µL was used for the lipid extraction of plasma samples.

### 2.7. Calculation of Fold Changes and Hypothesis Testing

**Figure 1:**
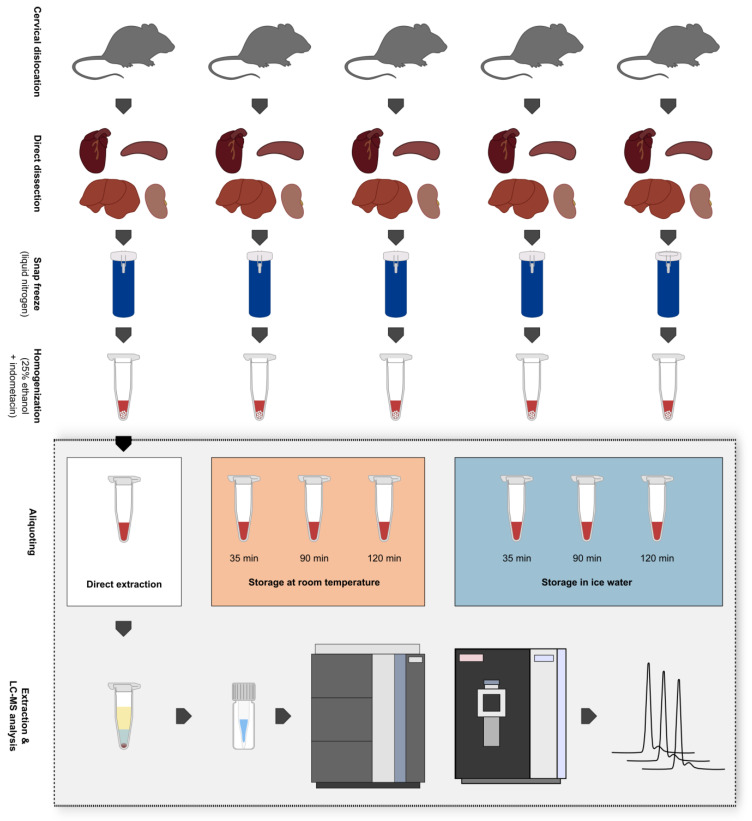
Overview of the sampling procedure, creation of biological replicates, and experimental setup. Five mice were sacrificed via cervical dislocation and heart, spleen, liver, and kidney samples were immediately dissected afterwards. Tissue samples were snap-frozen in liquid nitrogen and stored at −80 °C until further processing. Samples were then homogenized via wet bead milling using 25% ethanol + 10 µM indometacin as extraction solvent. After the storage of tissue homogenates at different conditions for varying periods, they were subjected to liquid–liquid extraction and were analyzed in a randomized order via ultra-high-performance liquid chromatography-high-resolution mass spectrometry (UHPLC-HRMS). Storage conditions: 1. Direct extraction after tissue homogenization (t_0_); 2. Storage at room temperature (RT) for max. 120 min; 3. Storage in ice water (IW) for max. 120 min.

For each mouse (*n* = 5), organs were dissected and separately homogenized. In this manner, four homogenates per mouse and thus five homogenates per tissue type were created. From each homogenate, samples for storage under investigated conditions and corresponding time points were aliquoted (Figure 1). Fold changes were calculated for samples that originated from the same homogenate and that were stored under the same condition. On that basis, five fold changes per time point were computed, which were then used to determine the arithmetic mean and the standard deviation. A fold increase ≥1.3 or decrease ≤0.7 compared to t_0_ was required to be considered a change in our evaluation.

Additionally, hypothesis testing was performed in GraphPad Prism 9.2.0 (GraphPad Software, San Diego, CA, USA) by applying a two-way ANOVA. Fold changes obtained at 35 min, 90 min, and 120 min were compared to 0 min (direct extraction, t_0_). A correction for multiple comparisons was performed via the use of Dunnett’s test. Statistical significance is presented as follows: * *p* < 0.05, ** *p* < 0.01, *** *p* < 0.001, and **** *p* < 0.0001. Data used for hypothesis testing and calculated significances can be found in the Supplementary Material (Data S1).

## 3. Results and Discussion

### 3.1. Lipid Stability in Tissue Homogenates

Liver, kidney, heart, and spleen homogenates of five mice were either stored at RT or in IW. Extraction and analysis of homogenates were performed directly (0 min) and after storage for 35 min, 90 min, and 120 min for both conditions (Figure 1). After tissue homogenates were stored for varying times under different conditions, samples were treated equally and were measured in a randomized order. Therefore, we expected that only these factors had a relevant influence on the results. To assess the stability of the homogenized tissues, multiple lipid class ratios were calculated as previously described [16] with the addition of further lipid ratios (10 ratios in total). A fold increase ≥1.3 or decrease ≤0.7 compared to direct extraction was required to be considered a change in our evaluation.

**Figure 2:**
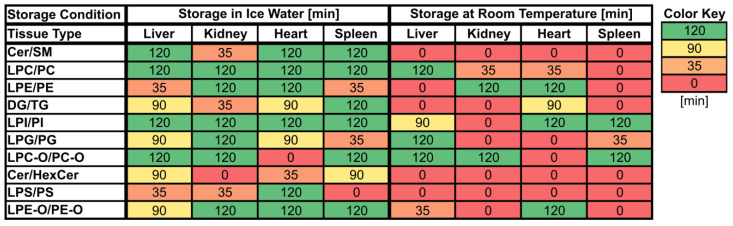
Maximum storage times of various tissue homogenates (liver, kidney, heart, spleen) at room temperature (RT) and in ice water (IW) until changes (≥30% absolute relative change compared to direct extraction) in lipid class ratios became visible. Values are given in minutes [min] and cells are colored based on the corresponding values.

**Figure 3:**
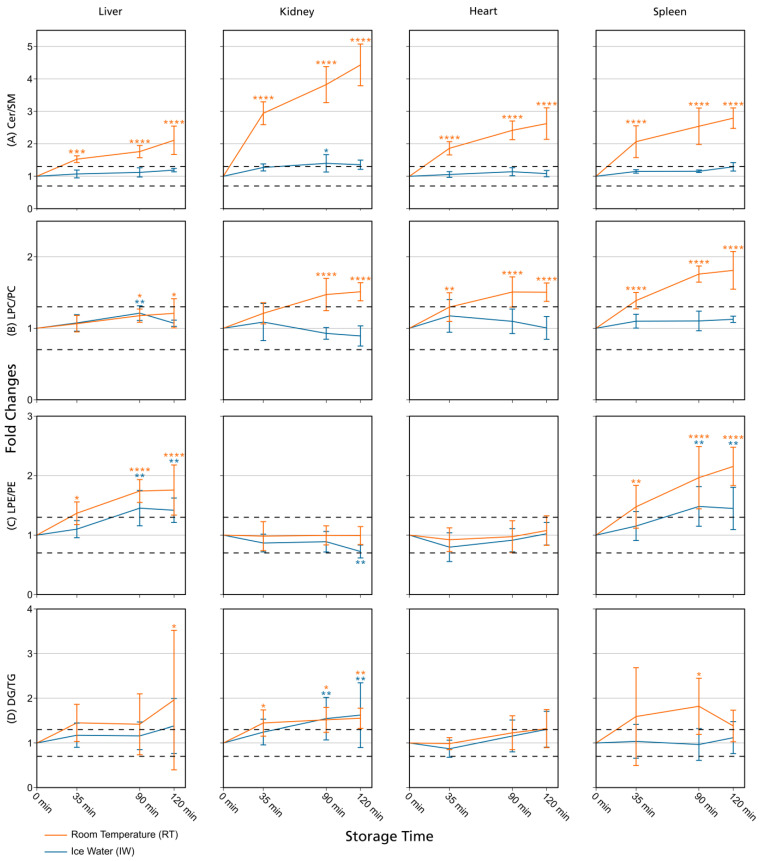
Lipid class ratios of homogenized liver, kidney, heart, and spleen samples either stored at room temperature (RT) or in ice water (IW) for specified times. Tissue types are organized in columns and lipid class ratios are depicted in the following rows: (**A**) Cer/SM; (**B**) LPC/PC; (**C**) LPE/PE; (**D**) DG/TG. The results are shown as fold changes of the ratios relative to their initial values at 0 min. Dashed lines indicate a relative lipid class ratio increase and decrease of 30%. Error bars represent corresponding standard deviations. Results are solely shown for lipids that passed additional filter criteria. * *p* < 0.05, ** *p* < 0.01, *** *p* < 0.001, and **** *p* < 0.0001 (see Section 2.7). Cer: ceramides; DG: diglycerides; LPC: lysophosphatidylcholines; LPE: lysophosphatidylethanolamines; PC: phosphatidylcholines; PE: phosphatidylethanolamines; SM: sphingomyelins; TG: triglycerides.

**Figure 4:**
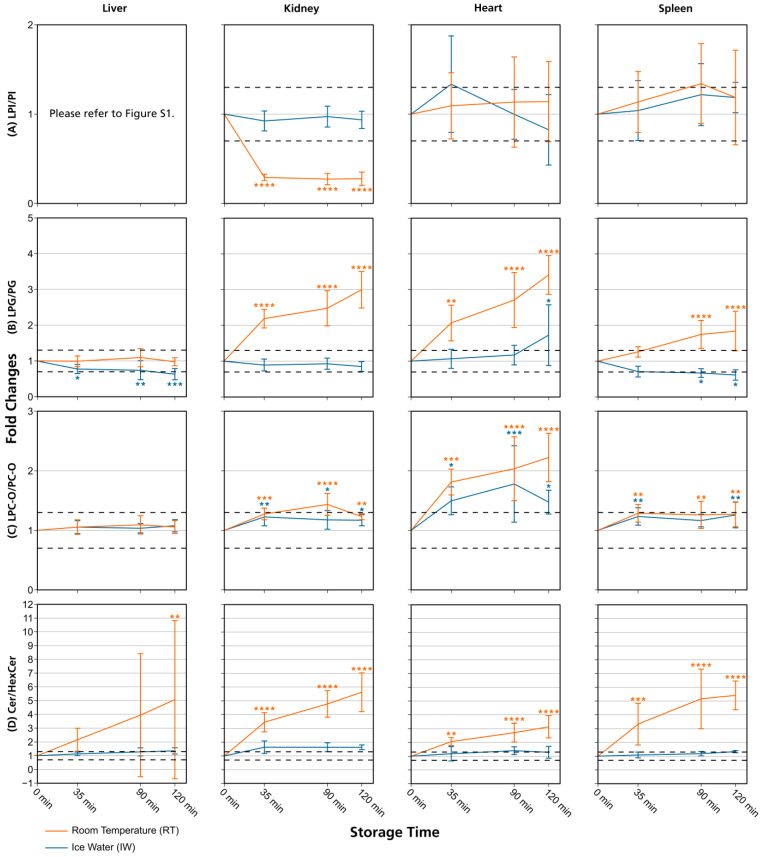
Lipid class ratios of homogenized liver, kidney, heart, and spleen samples either stored at room temperature (RT) or in ice water (IW) for specified times. Tissue types are organized in columns and lipid class ratios are depicted in the following rows: (**A**) LPI/PI; (**B**) LPG/PG; (**C**) LPC-O/PC-O; (**D**) Cer/HexCer. The results are shown as fold changes of the ratios relative to their initial values at 0 min. Dashed lines indicate a relative lipid class ratio increase and decrease of 30%. Error bars represent corresponding standard deviations. Results are shown for lipids that passed additional filter criteria with the only exception of LPI/PI liver data, as no LPIs remained after filtering. * *p* < 0.05, ** *p* < 0.01, *** *p* < 0.001, and **** *p* < 0.0001 (see Section 2.7). Cer: ceramides; HexCer: hexosylceramides; LPC-O: ether-linked lysophosphatidylcholines; LPG: lysophosphatidylglycerols; LPI: lysophosphatidylinositols; PC-O: ether-linked phosphatidylcholines; PG: phosphatidylglycerols; PI: phosphatidylinositols.

The use of fold changes is well established in lipidomics and metabolomics studies [16,30], and fold change thresholds were set because the maximum measurement error for the evaluated lipids, as depicted by their respective QC RSDs, was 20%. Thresholds of 20–30% are commonly used for technical variance and since we expected the data to be influenced by biological variability (see Section 2.7), we applied a 30% threshold in our evaluation. Additionally, we performed hypothesis testing and calculated *p*-values for the fold changes at 35 min, 90 min, and 120 min (see Section 2.7 and Data S1). In general, the results obtained by evaluating fold changes passing a certain threshold and hypothesis testing were comparable and exhibited few differences. However, the application of fold change thresholds proved to be more conservative as this approach was more sensitive to changes in lipid class ratios (Figure 2, Figures S11 and S12), which improved the conclusiveness of the data. For this reason, the focus of our evaluation lies on the assessment of fold changes exceeding thresholds, but significant changes are also shown in Figure 3 and Figure 4, Figures S1 and S2, and calculated significances can be found in the Supplementary Material.

Overall, lipid class ratios obtained by sample storage in IW were more stable compared to storage at RT. Almost all lipid class ratios, i.e., 93%, were unchanged (fold increases <1.3 or decreases >0.7) for up to 35 min when homogenates were stored in IW (Figure 2, Figure 3 and Figure 4, Figures S1 and S2).

**Figure 5:**
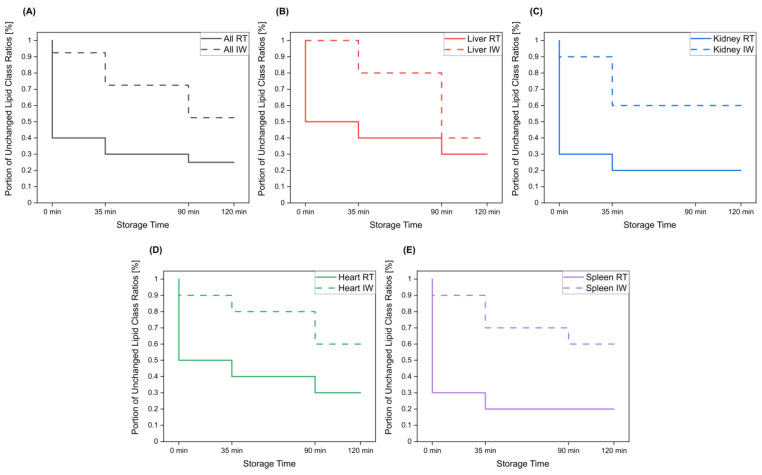
The portion of lipid class ratios that remained unchanged (<30% relative change compared to direct extraction) after storage of tissue homogenates for specified times at room temperature and in ice water. All investigated lipid class ratios were considered. Results are shown for (**A**) all investigated tissue homogenates, as well as for (**B**) liver, (**C**) kidney, (**D**) heart, and (**E**) spleen homogenates.

Lipid class ratios that were above the 1.3-fold increase limit after 35 min only included ceramides/hexosylceramides (Cer/HexCer, Figure 4D) ratios in kidney, ether-linked lysophosphatidylcholines/ether-linked phosphatidylcholines (LPC-O/PC-O, Figure 4C) ratios in heart, and lysophosphatidylserines/phosphatidylserines (LPS/PS, Figure S2) ratios in spleen homogenates. We consider LPS/PS ratios to be stable for 120 min in heart homogenates as the increase at 35 min was only minor and the two following time points were within the limit. At 90 min, 73% of investigated lipids remained unaltered (Figure 2 and Figure 5).

However, lipid class ratios that exhibited a fold increase >1.3 after 90 min storage in IW encompass ceramides/sphingomyelins (Cer/SM, Figure 3A), diglycerides/triglycerides (DG/TG, Figure 3D), and LPS/PS ratios in kidney homogenates, as well as lysophosphatidylethanolamines/phosphatidylethanolamines (LPE/PE, Figure 3C), and LPS/PS ratios in liver homogenates. In spleen samples, LPE/PE and lysophosphatidylglycerols/phosphatidylglycerols (LPG/PG, Figure 4B) ratios were altered after 90 min storage in IW, while Cer/HexCer ratios were solely increased in heart samples. After 120 min, 55% of investigated lipid class ratios stayed stable in IW. Most fold changes >1.3 at this point were related to liver homogenates, i.e., increased DG/TG, LPG/PG, Cer/HexCer, and lysophosphatidylethanolamines/ether-linked phosphatidylethanolamines (LPE-O/PE-O) ratios.

Approximately 40% of lipid class ratios remained stable in liver homogenates stored in ice water for 120 min (Figure 2 and Figure 5). Moreover, kidney homogenates possessed the fastest progressing changes, as 40% of lipid class ratios were already increased after 90 min storage time (Figure 2 and Figure 5). Further lipid class ratios that displayed fold increases >1.3 after 120 min storage in IW were DG/TG and LPG/PG ratios in heart samples, as well as Cer/HexCer ratios in spleen samples. Lysophosphatidylcholines/phosphatidylcholines (LPC/PC, Figure 3B) and lysophosphatidylinositols/phosphatidylinositols (LPI/PI, Figure 4A and Figure S1) ratios were the most stable ones, as no changes were registered even after 120 min of storage in IW.

In contrast, investigated lipid class ratios were mostly >1.3 fold increased when stored at RT (Figure 2, Figure 3 and Figure 4, Figures S1 and S2). The portion of stable ratios decreased to 40% after 35 min of storage (Figure 2 and Figure 5). Cer/SM, Cer/HexCer, and LPS/PS ratios were already above the threshold after 35 min storage in all tissue homogenates and all three ratios showed large fold increases, e.g., >4 for Cer/SM ratios in kidney samples at 120 min (Figure 3A). Therefore, they seem suitable to sensitively indicate lipolytic activity and sample stability at RT. For Cer/SM ratios, this is also in accordance with the conclusions made by Krautbauer et al. [16]. Although the standard deviations for Cer/HexCer and LPS/PS ratios were extremely high in some homogenates (Figure 4 and Figure S2), most Cer/HexCer and LPS/PS ratios exhibited significant changes after 35 min, except for Cer/HexCer ratios in liver and LPS/PS ratios in heart homogenates.

Increased standard deviations might have been caused by inter-individual differences of the study animals (see Section 2.7) or—as in the case of Cer/HexCer ratios—by multiple degradation processes that can release Cer. Besides the possible degradation of SM to Cer, the degradation of HexCer to Cer was previously described by [31,32]. Therefore, both degradation processes might take place simultaneously and affect Cer concentrations and the corresponding ratios (Cer/SM, Cer/HexCer). Beyond that, large standard deviations in LPS/PS ratios might be caused by the fact that LPS/PS data were not additionally filtered (see Section 2.6), which was also the case for LPE-O/PE-O data. This was because applying further filter criteria resulted in the deletion of LPS and LPE-O results in these tissues. Nevertheless, we included this data for LPS/PS and LPE-O/PE-O ratios (Figure S2) as we wished to provide the most comprehensive picture of lipid changes.

DG/TG ratios at RT were unstable in all investigated tissue homogenates, except in heart samples, in which DG/TG ratios stayed <30% relative change for up to 90 min. DG/TG ratios also possessed large standard deviations, particularly in liver and spleen samples, which additionally impairs the conclusiveness of the data. This might be linked to the fact that DGs can be further degraded to monoglycerides and fatty acids. However, hypothesis testing revealed significant changes for the 120 min and 90 min points in liver and spleen homogenates, respectively. In conjunction with the data previously reported by Krautbauer et al. [16], this suggests that DG/TG ratios are increasing in a time-dependent manner in liver and spleen homogenates when stored at RT. Moreover, LPI/PI ratios (Figure 4 and Figure S1) exhibited a relative decrease of approx. 70% for kidney homogenates that were stored at RT. Here, a closer inspection of the data (Figure S5 and Data S1) revealed that average LPI levels drop after 35 min, while average PI levels are hardly changing. This decreasing trend is special as it was not observed to that extent for any other lipid class ratio, and it was not present in kidney homogenates that were stored in IW. This finding might be linked to LPI’s role as a bioactive lipid messenger and its diverse ways of enzymatic degradation [33].

Ultimately, only 30% and 25% of investigated lipid class ratios were stable at RT up until 90 min and 120 min, respectively. The most and the fastest progressing alterations under this storage condition were found in kidney and spleen homogenates, while liver and heart homogenates were slightly more stable (Figure 2 and Figure 5). These alterations were also visible in the overall lipid profiles, with the most prominent changes detected in spleen homogenates, as depicted in Figure S13. It should be noted that the data are presented as mean ratios of individual lipids, which may be differently sensitive to temperature. However, most lipids belonging to one lipid class showed similar changes, which are displayed in Figures S3–S10. Interestingly, we could identify some differences between saturated and unsaturated lipids, e.g., unsaturated LPC showed more prominent increasing trends in kidney and spleen homogenates under both storage conditions (Figures S3, S4, S9 and S10). Nonetheless, most investigated lipids were unsaturated and the results for the comparison of saturated vs. unsaturated lipids were mostly ambiguous.

Taking all of our findings into account, swift sample extraction (≤35 min) of tissue homogenates in IW is recommended. A prolonged extraction time of 90 min seems to be acceptable, but researchers should be aware of the changes in lipid profiles that might occur, e.g., alterations in LPS/PS, Cer/HexCer, and LPE/PE ratios. Sample preparation of homogenized tissue samples at ambient temperature should be avoided.

### 3.2. Comparison with Previously Published Studies

Lipid class ratios for assessing sample stability in tissue homogenates at ambient temperature have been investigated before by Krautbauer and colleagues [16]. In their study, liver, brain, lung, heart, and spleen samples were analyzed, and thus three tissue types (liver, heart, and spleen) are overlapping with the hereby-presented study. However, the application of lipid class ratios to monitor lipid stability in kidney samples is exclusive to our study and was not studied before. We could verify the results by Krautbauer and colleagues (Cer/SM, LPC/PC, LPE/PE, DG/TG ratios at RT) [16], but also extended the investigated lipid classes and lipid class ratios (LPI/PI, LPG/PG, LPC-O/PC-O, Cer/HexCer, LPS/PS, LPE-O/PE-O). For the mutually investigated tissue types and lipid class ratios, we were able to reproduce most of the results from Krautbauer and colleagues [16]. We could also confirm that Cer/SM ratios exhibit the largest increases (at RT without stabilization) and that tissues differ in lipolytic activity. Concerning Cer/SM and LPC/PC ratios, all results could be reproduced. There were only a few exceptions where we noted different results. In contrast to Krautbauer et al. [16], we observed no relative change ≥30% compared to t_0_ for LPE/PE ratios in heart samples, although an increase of approx. 75% at 2 h was stated before. Moreover, in the liver samples that they investigated, only a slightly increasing trend for LPE/PE ratios at 2 h was revealed [16], whereas we could detect a clear increase of approx. 75% relative change, which also appeared significant after hypothesis testing. It is important to note that our results on the 2 h point exhibit a comparatively large standard deviation, which impairs their conclusiveness. This might be related to the experimental setup, as replicate samples (*n* = 5) were not prepared from one homogenate pool but were created from individual homogenates, allowing for possible metabolic differences. Lastly, Krautbauer et al. showed an approximate 60% increase in DG/TG ratios for heart tissue homogenates [16] but we observed just a slight increase of approx. 30% at 2 h. However, this change was not significant after performing hypothesis testing, in contrast to DG/TG ratio changes in liver and spleen homogenates that were revealed to be significant for the 120 min and 90 min points, respectively. The observed differences might arise from differences in the analyzed lipid profiles, as the investigated analytes as well as their total number per lipid class differ between the studies.

As indicated in the introduction, there are multiple challenges during the sample preparation for tissue lipidomics, including the choice of extraction solvent or technique of homogenization. Besides affecting the recovery rate of lipids, the extraction solvent will most likely also influence the stability of the resulting homogenate. In this study, 25% ethanol with the addition of 10 µM indometacin was applied and the lipid stability shown in our study might depend on this extraction solvent as well as the indometacin. We still consider our results transferable, because of the close fit of our results for homogenates which were stored at RT to the results by Krautbauer and colleagues, who used 50% methanol as extraction solvent [16].

## 4. Conclusions

We conclude that lipid stability in tissue homogenates during sample extraction can be improved by working under cooled conditions and as fast as possible (≤35 min). Our results reveal that short-term storage (≤120 min) of tissue homogenates at ambient temperature causes considerable changes in lipid profiles. Within 35 min, 60% of investigated lipid class ratios exhibited relative changes >30% compared to direct extraction. In contrast, storage in IW stabilized investigated lipid class ratios in liver, kidney, heart, and spleen homogenates, as approx. 93% of them remained <30% relative change after 35 min. Thus, homogenate extraction in IW is recommended, which is also easy to implement. It also reassures the results from previous tissue lipidomics studies, as storage under cooled conditions is a standard procedure to improve lipid stability [17]. If sample extraction takes more time, researchers should be aware of the lipid classes that are prone to changes. Nevertheless, the addition of SDS to tissue homogenates still seems to be superior in the cases where it does not interfere with the analysis, as it effectively stabilizes lipid class ratios for an extended period (>120 min) [16]. The here-presented investigation demonstrates the importance and impact of pre-analytical factors, and that storage in ice water is useful to improve lipid stability in tissue homogenates for a short time.

## Data Availability

The data presented in this study are available in Supplementary Material, Data S1.

## Supplementary Materials

The following supporting information can be downloaded at: https://www.mdpi.com/article/10.3390/metabo13040504/s1, Figure S1: Time-dependent LPI/PI ratio changes in liver homogenates stored at room temperature (RT) and in ice water (IW); Figure S2: LPS/PS and LPE-O/PE-O ratios of homogenized liver, kidney, heart, and spleen samples either stored at room temperature (RT) or in ice water (IW) for specified times; Figure S3: Heatmap depicting lipid fold changes in liver homogenates stored at RT; Figure S4: Heatmap depicting lipid fold changes in liver homogenates stored in IW; Figure S5: Heatmap depicting lipid fold changes in kidney homogenates stored at RT; Figure S6: Heatmap depicting lipid fold changes in kidney homogenates stored in IW; Figure S7: Heatmap depicting lipid fold changes in heart homogenates stored at RT; Figure S8: Heatmap depicting lipid fold changes in heart homogenates stored in IW; Figure S9: Heatmap depicting lipid fold changes in spleen homogenates stored at RT; Figure S10: Heatmap depicting lipid fold changes in spleen homogenates stored in IW; Figure S11: Heatmap depicting maximum storage times of tissue homogenates until significant changes became visible; Figure S12: Portion of lipid class ratios that remained unchanged after storage of tissue homogenates for indicated times; Figure S13: Pie charts illustrating the portions of lipid class signals measured in tissue homogenates; Data S1: Lipid data, fold changes data, hypothesis testing data, and method details.
