# Supplementary material for: Effects of Different Storage Conditions on Lipid Stability in Mice Tissue Homogenates
Source: Metabolites. 2023 Mar 31;13(4):504. doi: 10.3390/metabo13040504 (PMC10144362; doi:10.3390/metabo13040504)
Supplement: Supplementary file 1 [file metabolites-13-00504-s001.zip › Supplementary Material_Figures S1-S13_Rev_Cleaned_ED.pdf]

metabolites

MDPI

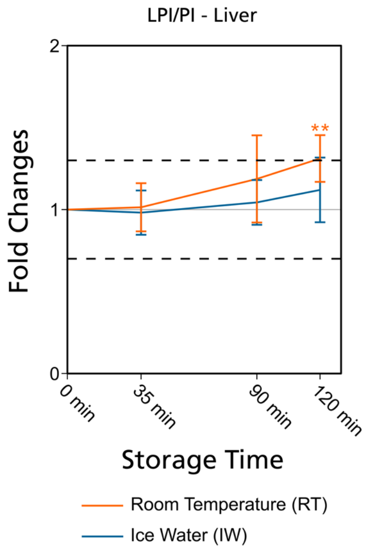

Figure S1. LPI/PI ratios of homogenized liver samples either stored at room temperature (RT) or in ice water (IW) for specified times. The results are shown as fold changes of the ratios relative to their initial values at 0 min. Dashed lines indicate a relative change of 30%. Error bars represent corresponding standard deviations. Results are shown for lipid data on which no additional filter criteria were applied. *$p < 0.05$, **$p < 0.01$, ***$p < 0.001$, and ****$p < 0.0001$ (see chapter 2.7.). LPI: lysophosphatidylinositols; PI: phosphatidylinositols.

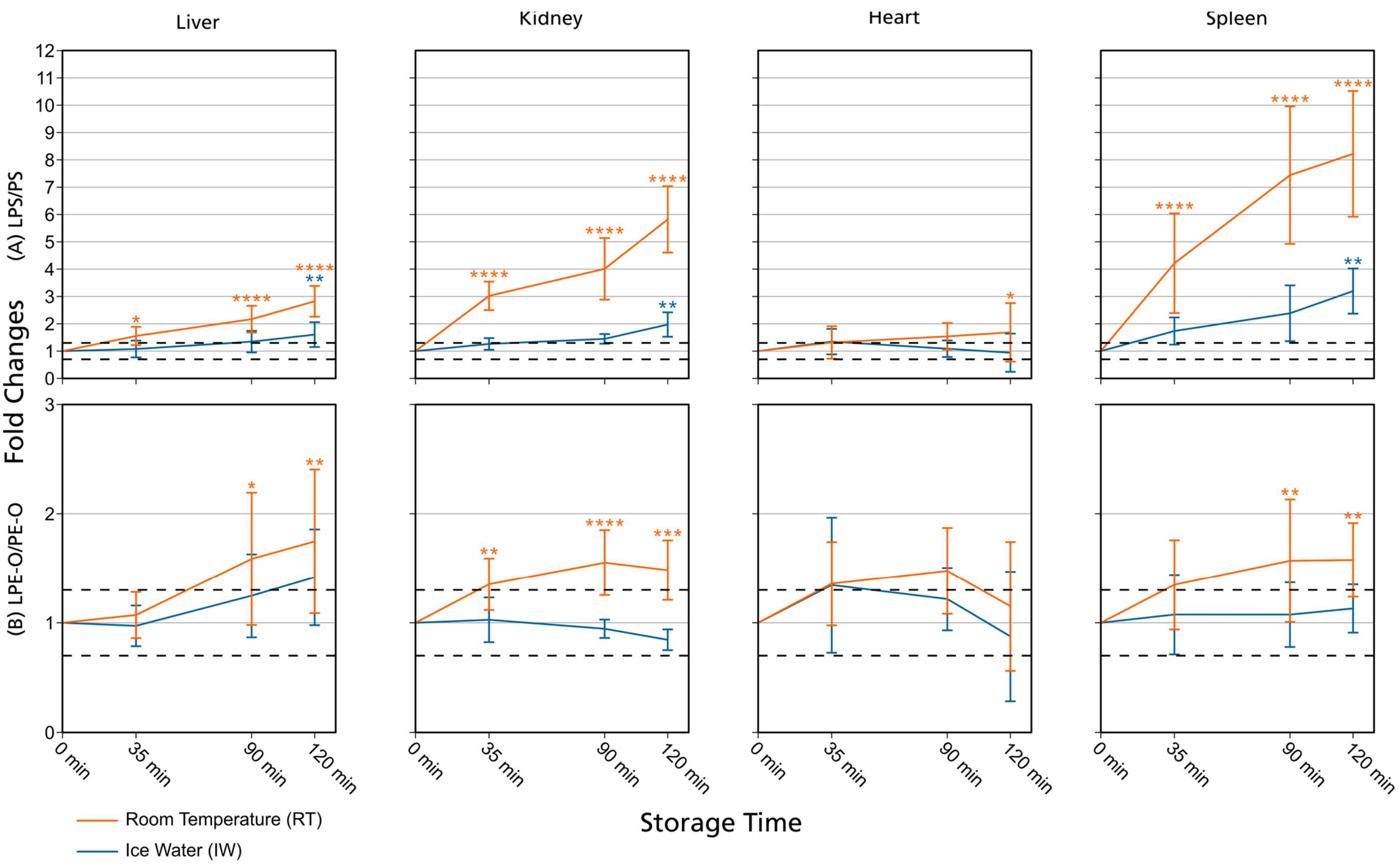

Figure S2. LPS/PS and LPE-O/PE-O ratios of homogenized liver, kidney, heart, and spleen samples either stored at room temperature (RT) or in ice water (IW) for specified times. Tissue types are organized in columns and lipid class ratios are depicted in the following rows: (A) LPS/PS; (B) LPE-O/PE-O. The results are shown as fold changes of the ratios relative to their initial values at 0 min. Dashed lines indicate a relative change of 30%. Error bars represent corresponding standard deviations. Results are shown for all evaluated lipids (no additional filter criteria were applied). *$p < 0.05$, **$p < 0.01$, ***$p < 0.001$, and ****$p < 0.0001$ (see chapter 2.7.). LPE-O: ether-linked lysophosphatidylethanolamines; LPS: lysophosphatidylserines; PE-O: ether-linked phosphatidylethanolamines; PS: phosphatidylserines.

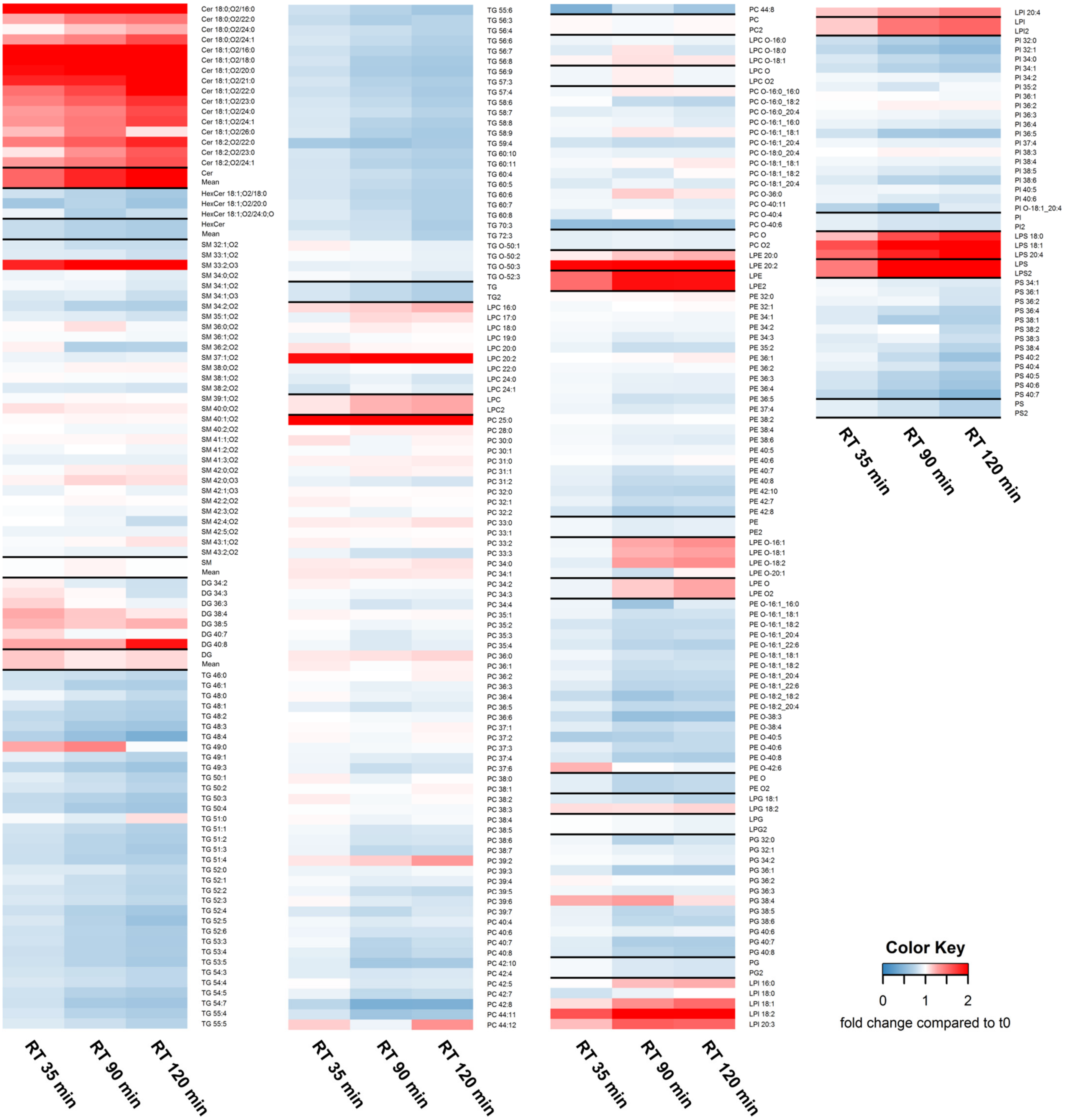

Figure S3. Heatmaps illustrating the data obtained for the lipid analysis of liver homogenates that were stored at room temperature (RT). The color key shows the fold changes of evaluated lipid signals at 35 min, 90 min, and 120 min relative to the intitial conditions (t_0). Average lipid class fold changes are indicated using black horizontal lines. Used data can be found in the supplementary material Data S1 ("Liver Lipid Fold Changes").

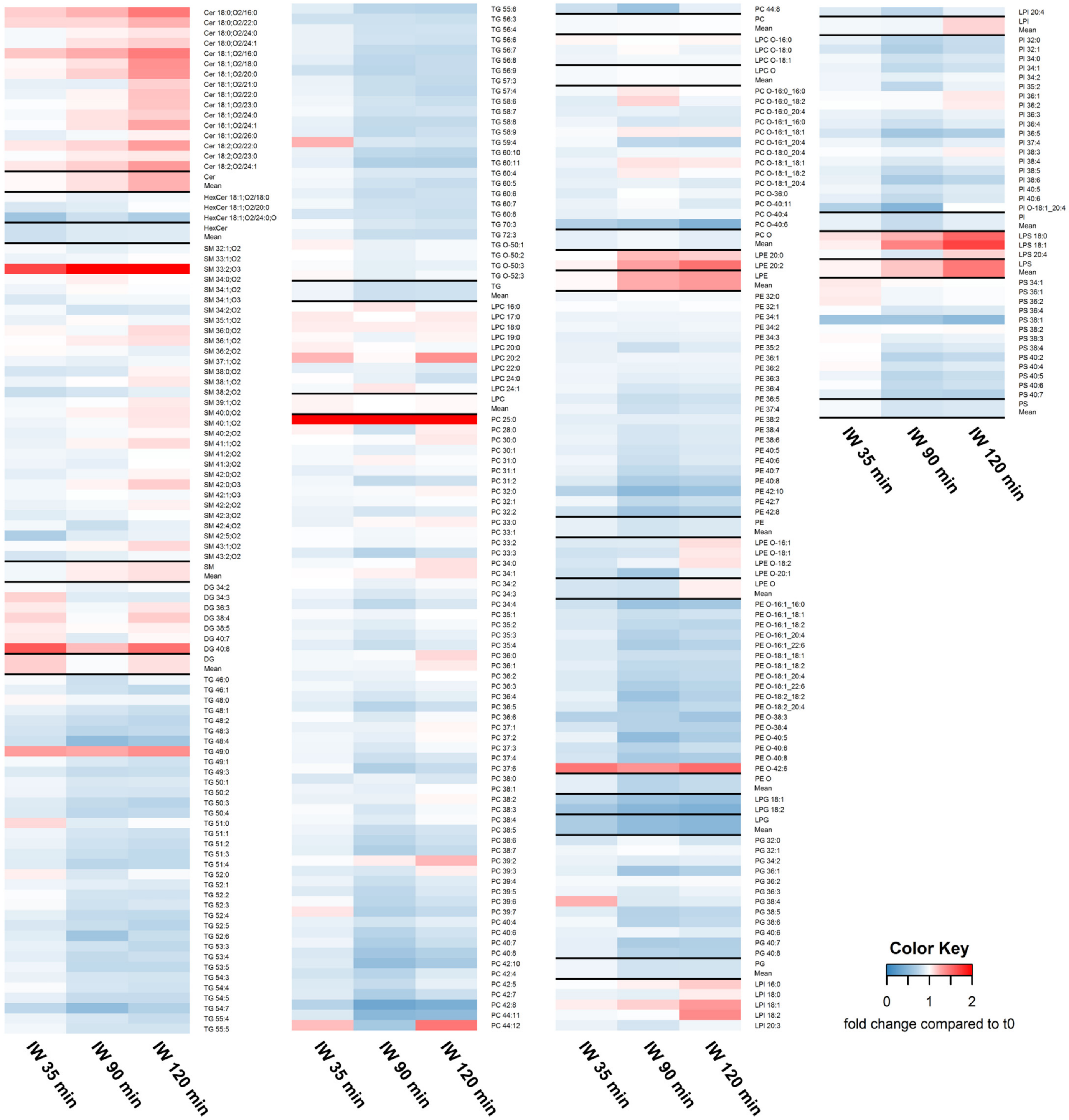

Figure S4. Heatmaps illustrating the data obtained for the lipid analysis of liver homogenates that were stored in ice water (IW). The color key shows the fold changes of evaluated lipid signals at 35 min, 90 min, and 120 min relative to the intitial conditions (t_0). Average lipid class fold changes are indicated using black horizontal lines. Used data can be found in the supplementary material Data S1 ("Liver Lipid Fold Changes").

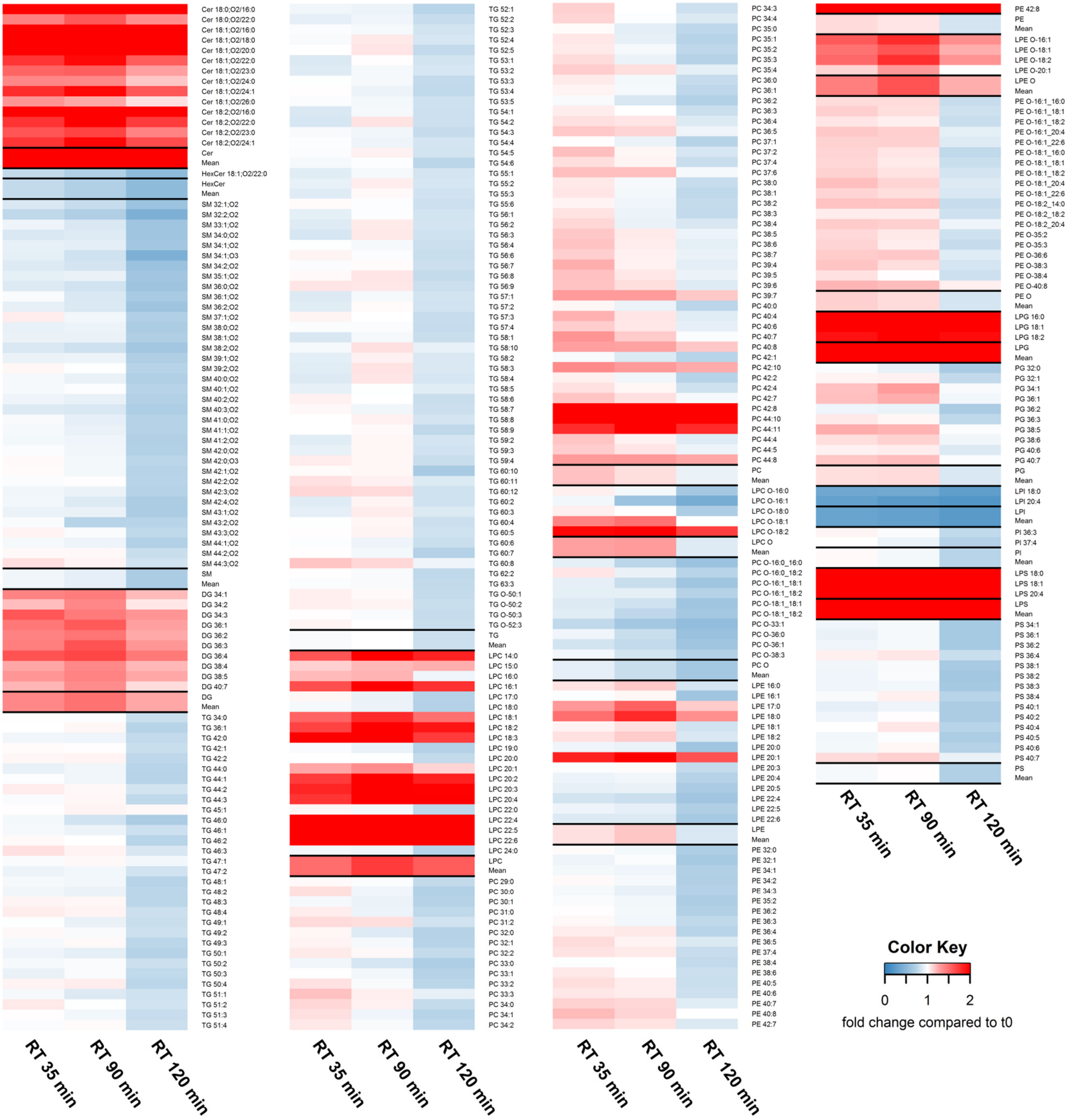

Figure S5. Heatmaps illustrating the data obtained for the lipid analysis of kidney homogenates that were stored at room temperature (RT). The color key shows the fold changes of evaluated lipid signals at 35 min, 90 min, and 120 min relative to the intitial conditions (t_0). Average lipid class fold changes are indicated using black horizontal lines. Used data can be found in the supplementary material Data S1 ("Kidney Lipid Fold Changes").

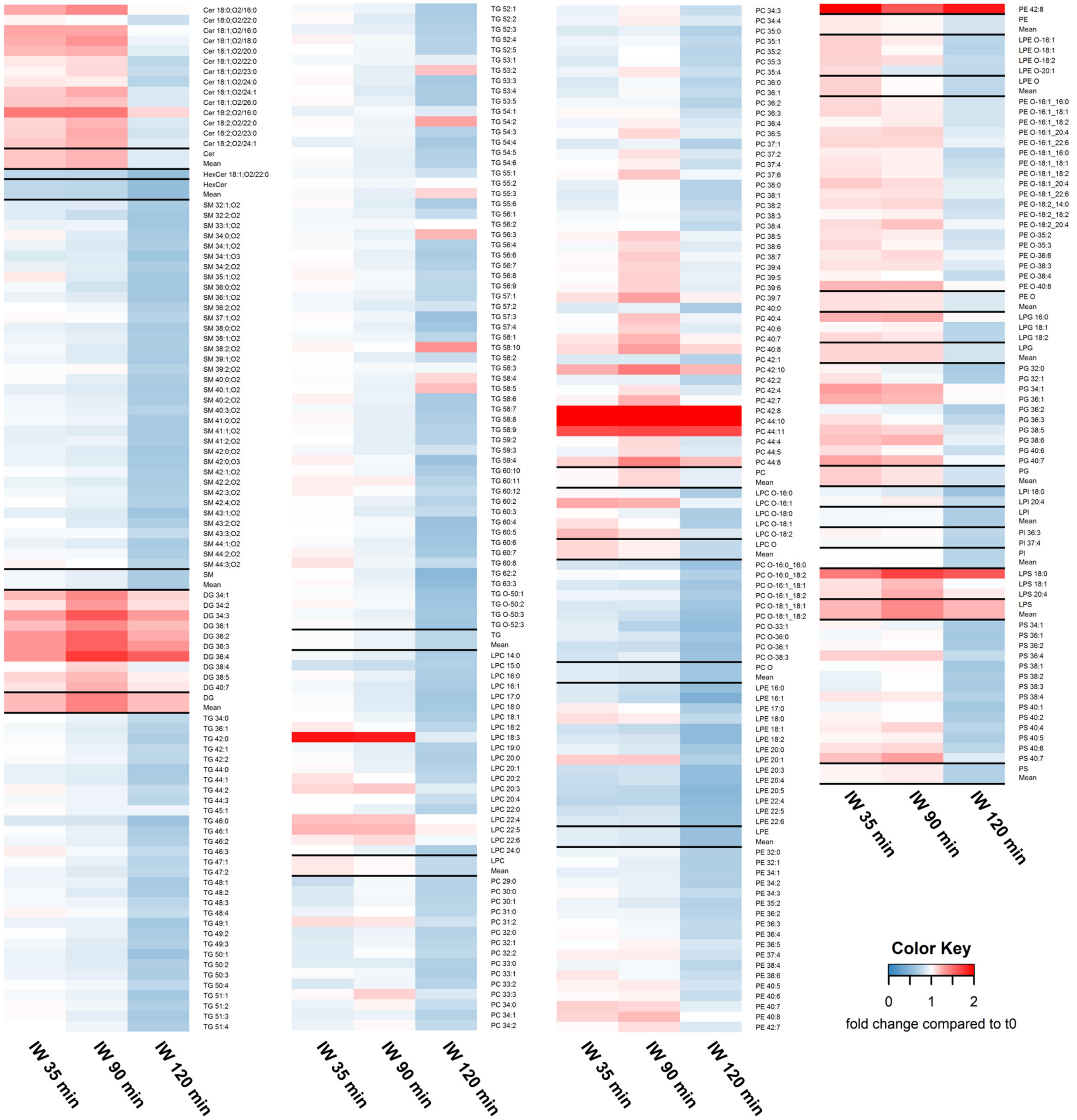

Figure S6. Heatmaps illustrating the data obtained for the lipid analysis of kidney homogenates that were stored in ice water (IW). The color key shows the fold changes of evaluated lipid signals at 35 min, 90 min, and 120 min relative to the intitial conditions (t_0). Average lipid class fold changes are indicated using black horizontal lines. Used data can be found in the supplementary material Data S1 ("Kidney Lipid Fold Changes").

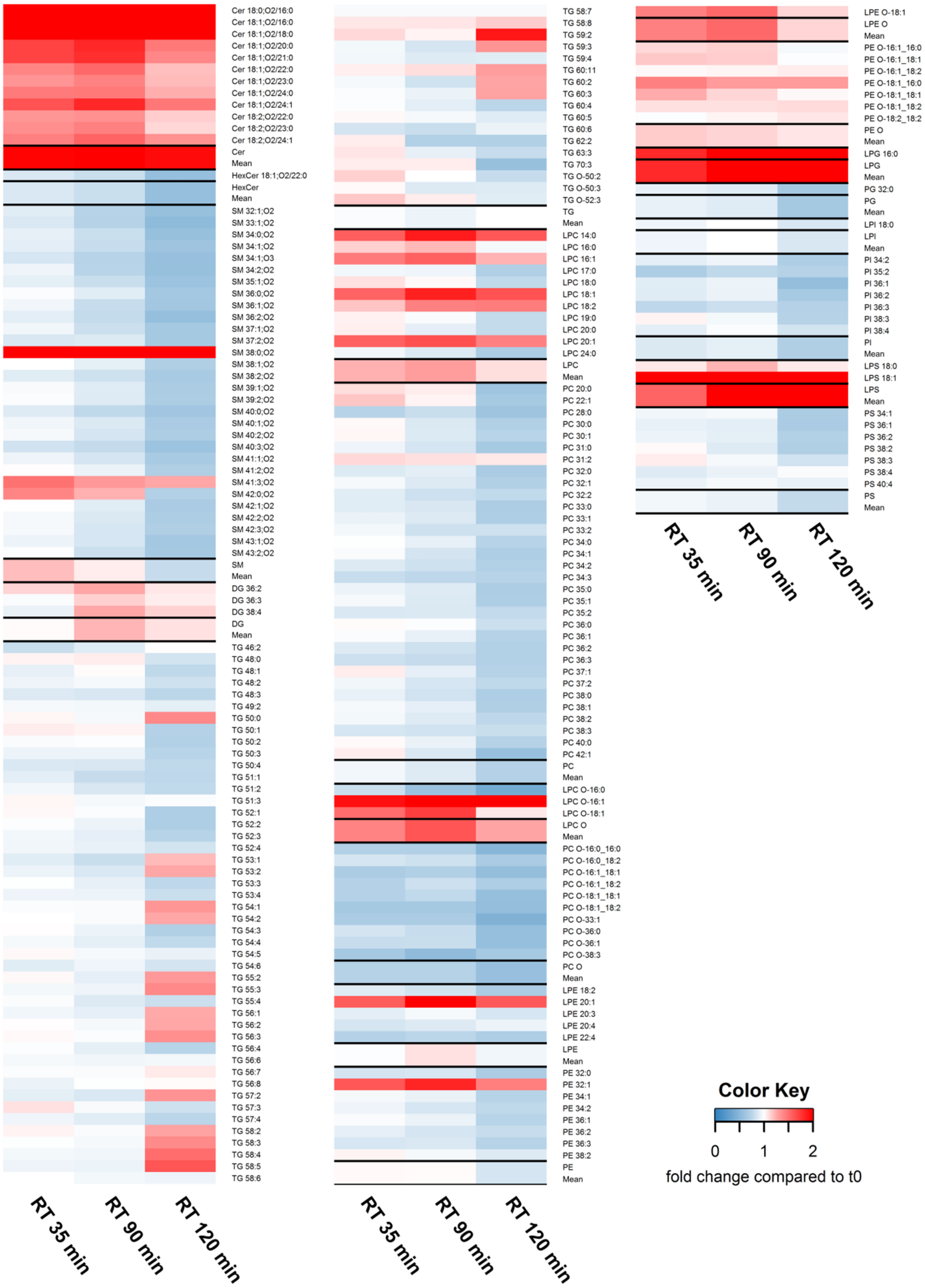

Figure S7. Heatmaps illustrating the data obtained for the lipid analysis of heart homogenates that were stored at room temperature (RT). The color key shows the fold changes of evaluated lipid signals at 35 min, 90 min, and 120 min relative to the intitial conditions (t_0). Average lipid class fold changes are indicated using black horizontal lines. Used data can be found in the supplementary material Data S1 ("Heart Lipid Fold Changes").

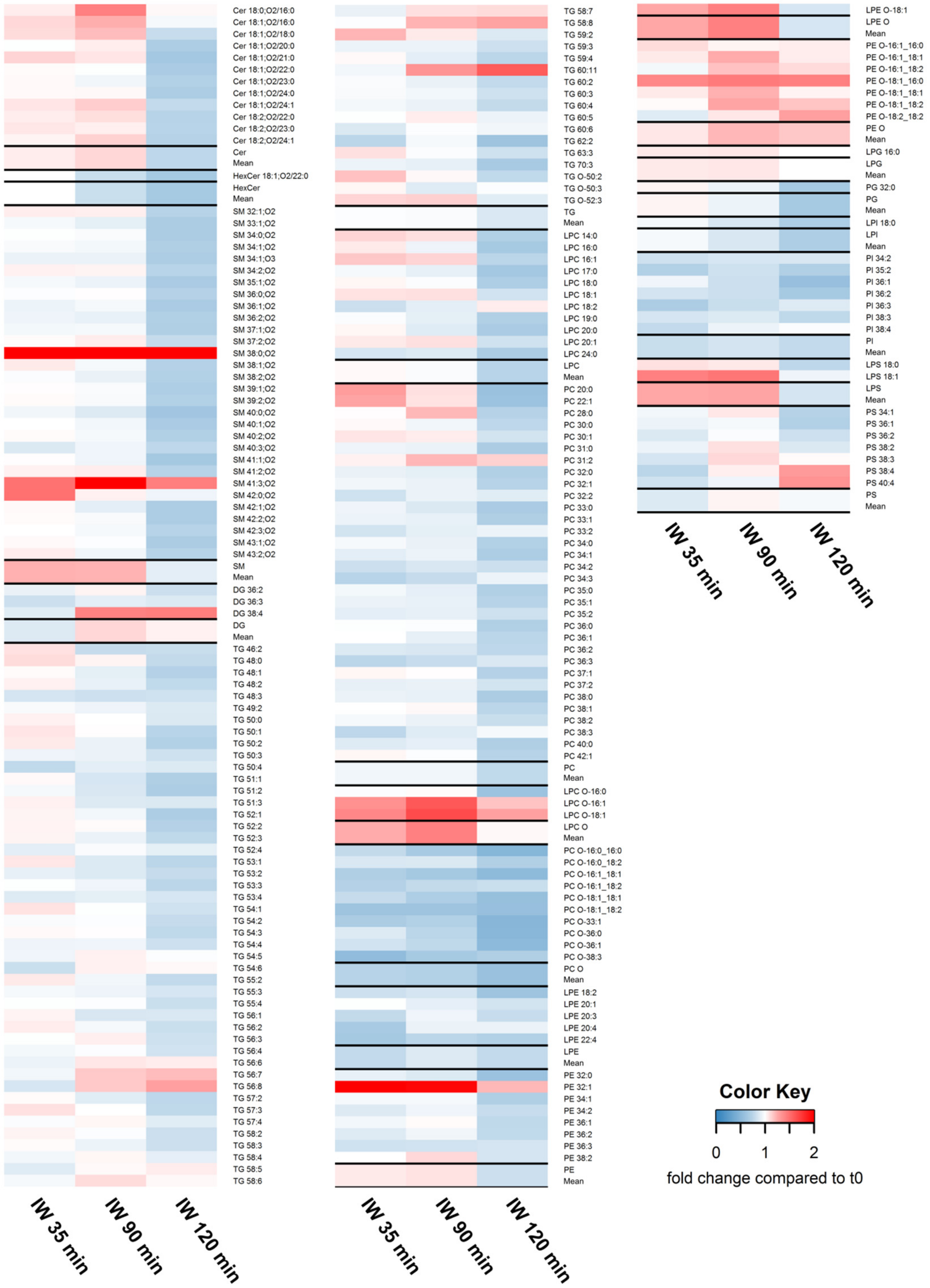

Figure S8. Heatmaps illustrating the data obtained for the lipid analysis of heart homogenates that were stored in ice water (IW). The color key shows the fold changes of evaluated lipid signals at 35 min, 90 min, and 120 min relative to the intitial conditions (t_0). Average lipid class fold changes are indicated using black horizontal lines. Used data can be found in the supplementary material Data S1 ("Heart Lipid Fold Changes").

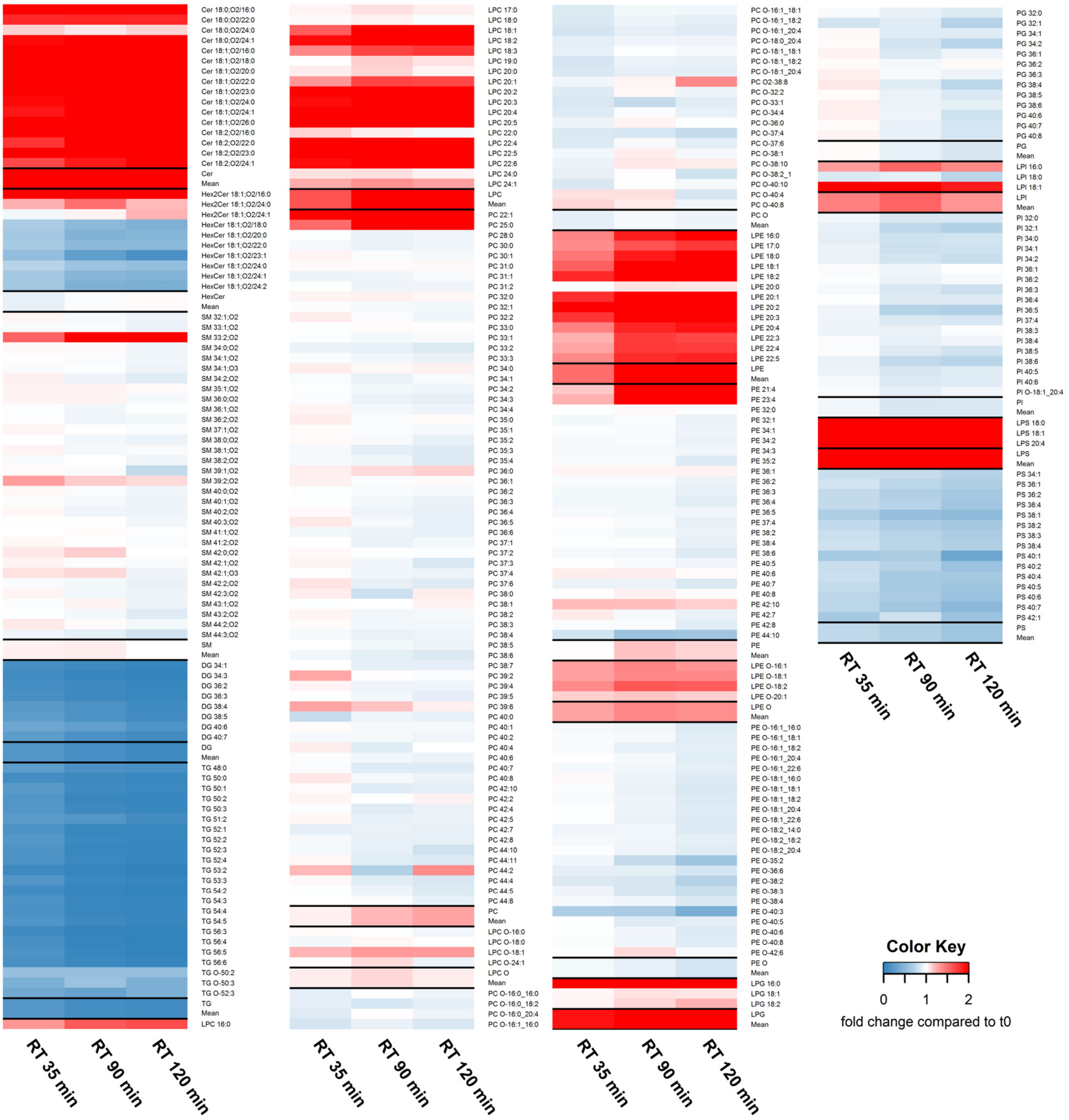

Figure S9. Heatmaps illustrating the data obtained for the lipid analysis of spleen homogenates that were stored at room temperature (RT). The color key shows the fold changes of evaluated lipid signals at 35 min, 90 min, and 120 min relative to the intitial conditions (t_0). Average lipid class fold changes are indicated using black horizontal lines. Used data can be found in the supplementary material Data S1 ("Spleen Lipid Fold Changes"). Interestingly, DG and TG lipids are showing a strong decrease, which was not observed in the other investigated tissue homogenates. However, as both DG and TG lipids were decreased, DG/TG ratios (Figure 2D) only showed fold increases < 2 and were comparable to the fold changes obtained for the measurement of the other tissue homogenates.

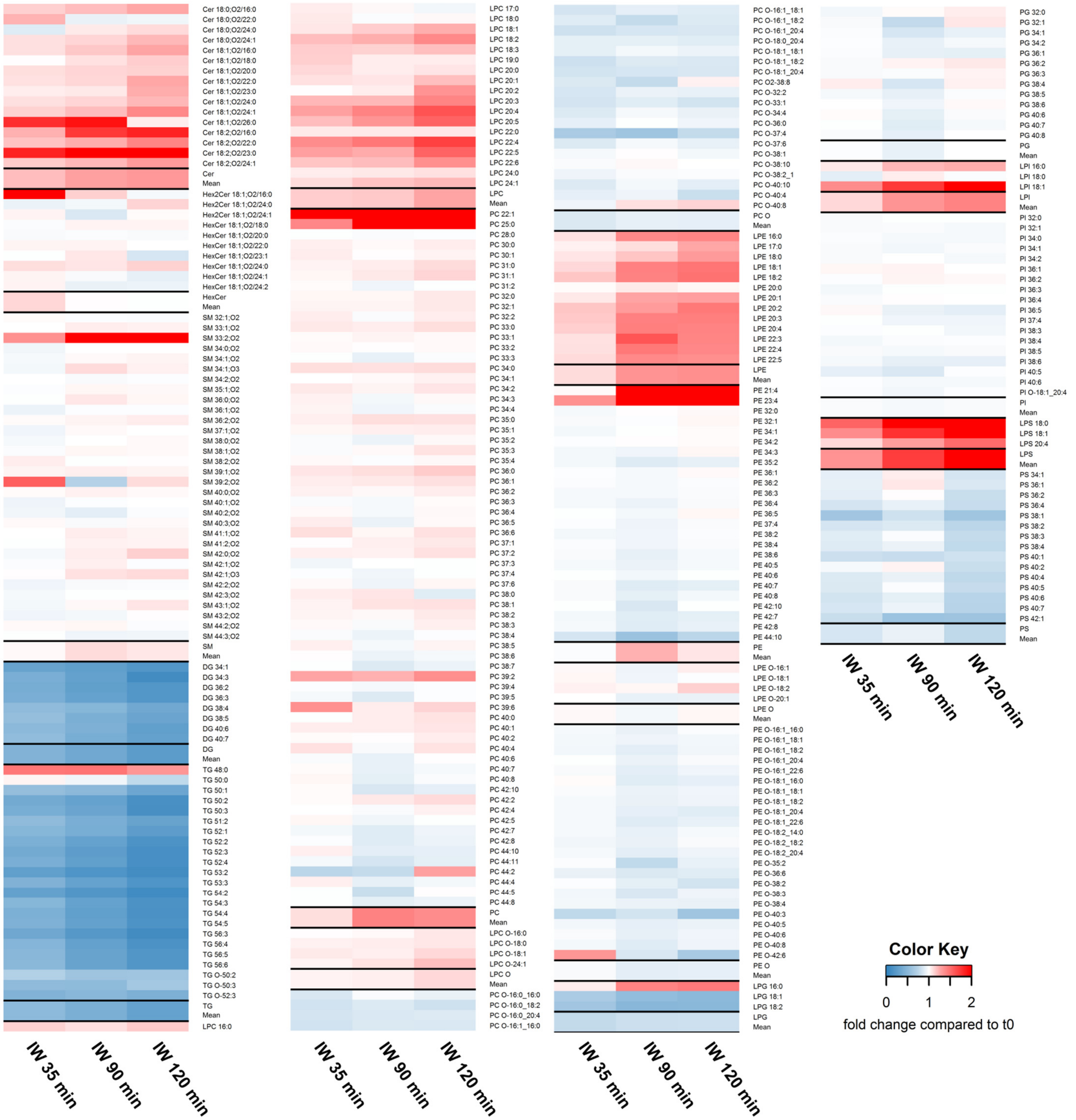

Figure S10. Heatmaps illustrating the data obtained for the lipid analysis of spleen homogenates that were stored in ice water (IW). The color key shows the fold changes of evaluated lipid signals at 35 min, 90 min, and 120 min relative to the intitial conditions (t_0). Average lipid class fold changes are indicated using black horizontal lines. Used data can be found in the supplementary material Data S1 ("Spleen Lipid Fold Changes"). Interestingly, DG and TG lipids are showing a strong decrease, which was not observed in the other investigated tissue homogenates. However, as both DG and TG lipids were decreased, DG/TG ratios (Figure 2D) only showed fold increases < 2 and were comparable to the fold changes obtained for the measurement of the other tissue homogenates.

Storage Condition	Storage in Ice Water [min]				Storage at Room Temperature [min]			
**Tissue Type**	**Liver**	**Kidney**	**Heart**	**Spleen**	**Liver**	**Kidney**	**Heart**	**Spleen**
**Cer/SM**	120	35	120	120	0	0	0	0
**LPC/PC**	35	120	120	120	35	35	0	0
**LPE/PE**	35	90	120	35	0	120	120	0
**DG/TG**	120	35	120	120	90	0	120	35
**LPI/PI**	120	120	120	120	90	0	120	120
**LPG/PG**	0	120	90	35	120	0	0	35
**LPC-O/PC-O**	120	0	0	0	120	0	0	0
**Cer/HexCer**	120	120	120	120	90	0	0	0
**LPS/PS**	90	90	120	90	0	0	90	0
**LPE-O/PE-O**	120	120	120	120	35	0	120	35

Color Key
120
90
35
0
[min]

Figure S11. Maximum storage times of various tissue homogenates (liver, kidney, heart, spleen) at room temperature (RT) and in ice water (IW) until significant changes ($p < 0.05$, $p < 0.01$, $p < 0.001$, or $p < 0.0001$, see chapter 2.7.) in lipid class ratios became visible. Values are given in minutes [min] and cells are colored based on the corresponding values. Comparison with Figure 3 reveals few differences which can also be observed in Figure S12.

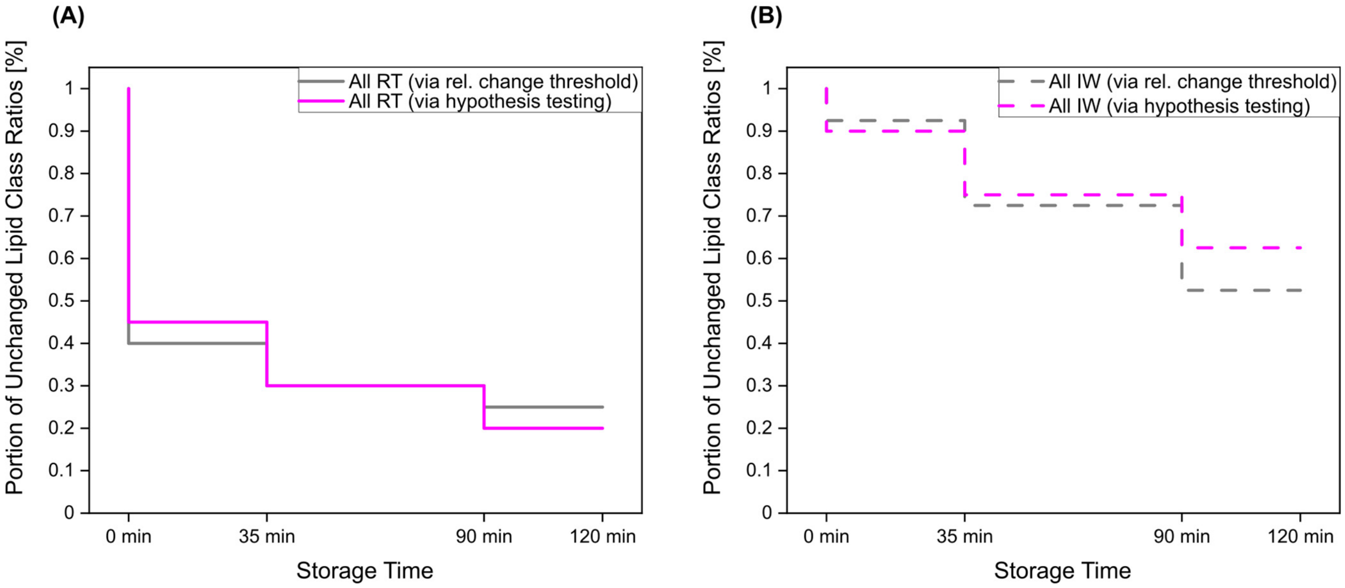

Figure S12. The portion of lipid class ratios that remained unchanged (< 30% relative change compared to direct extraction vs. unsignificant after hypothesis testing) after storage of tissue homogenates for specified times. All investigated lipid class ratios were considered. Results are shown for (A) storage at room temperature (RT) and (B) storage in ice water (IW).

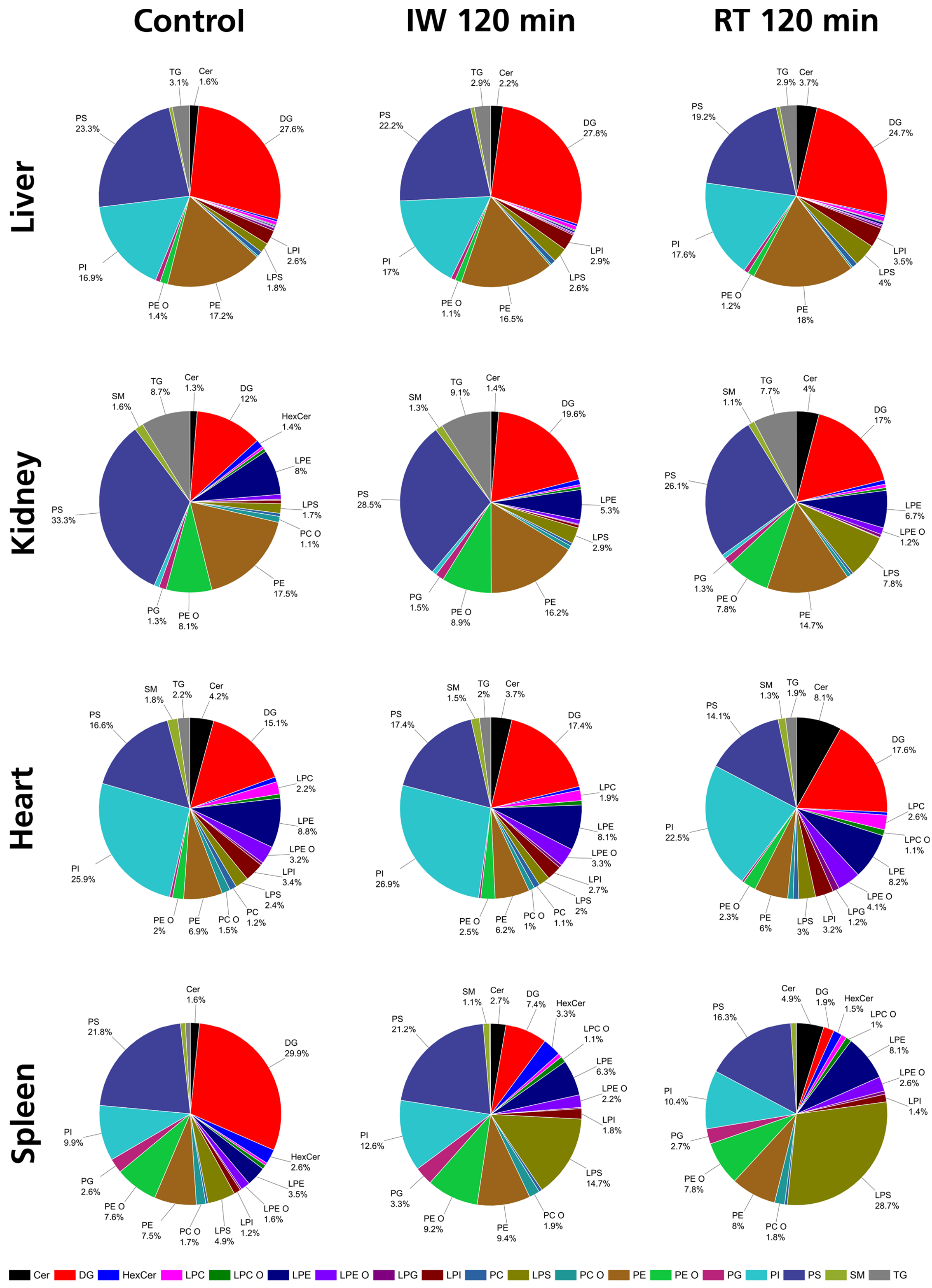

Figure S13. Pie charts illustrating the portions of lipid class signals measured in liver, kidney, heart, and spleen samples after direct extraction and storage for 120 min in ice water (IW) and at room temperature (RT). Portions were determined by calculating average lipid class signals that were summed afterwards. Then, average lipid class signals were divided by the sums. For better visualization, portions < 1% were not labelled. Spleen homogenates show the most prominent changes during storage under both conditions which is most likely linked to the strong decrease of DG (and TG) lipids that could be observed (see Figures S9-S10).
