# Supplementary figures and images for: Effects of Different Storage Conditions on Lipid Stability in Mice Tissue Homogenates
Source: Metabolites. 2023 Mar 31;13(4):504. doi: 10.3390/metabo13040504 (PMC10144362; doi:10.3390/metabo13040504)

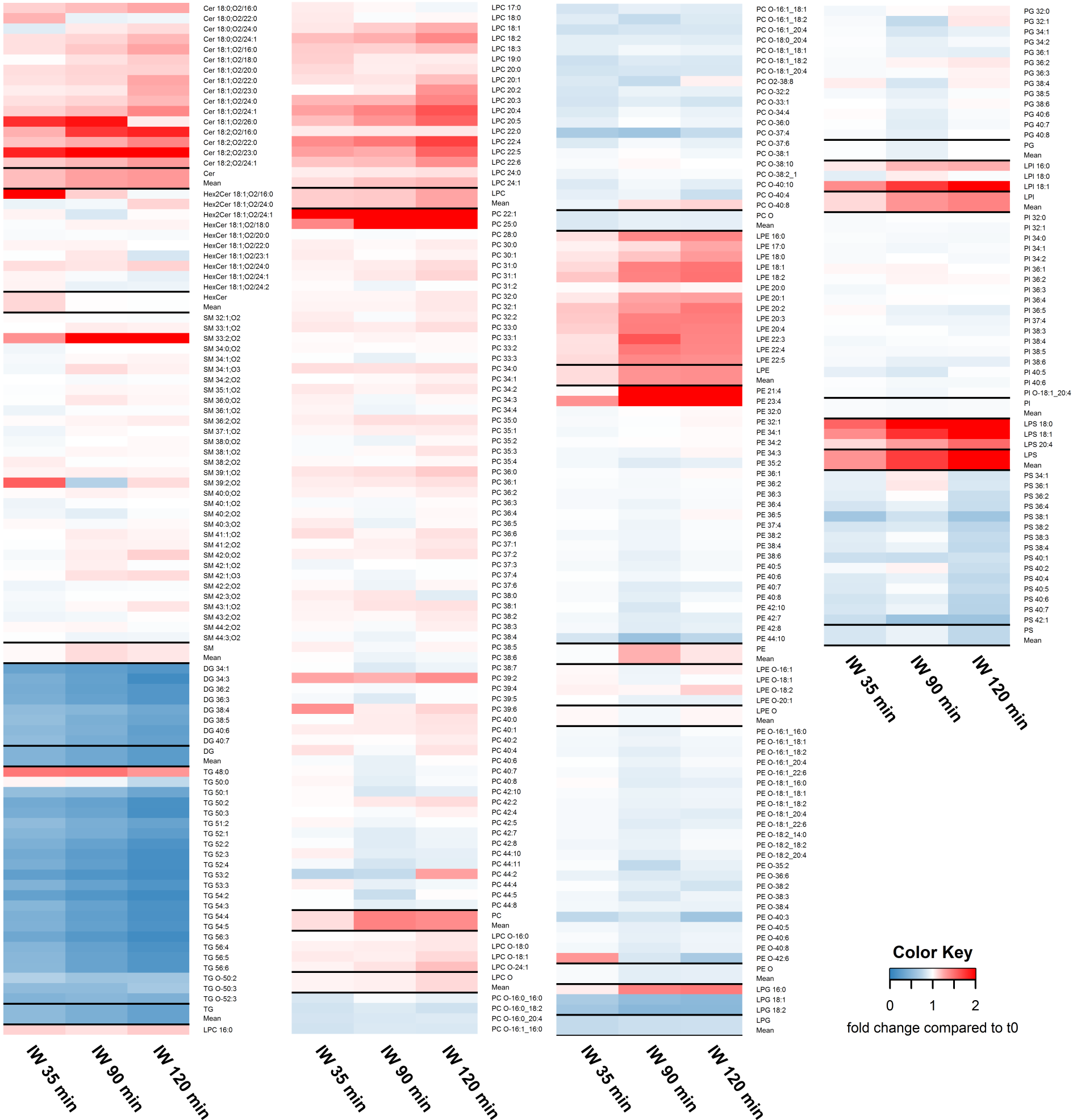

Supplement: Supplementary file 1 [file metabolites-13-00504-s001.zip › Figure S10_dpi300_2023-03-24_ED.png]

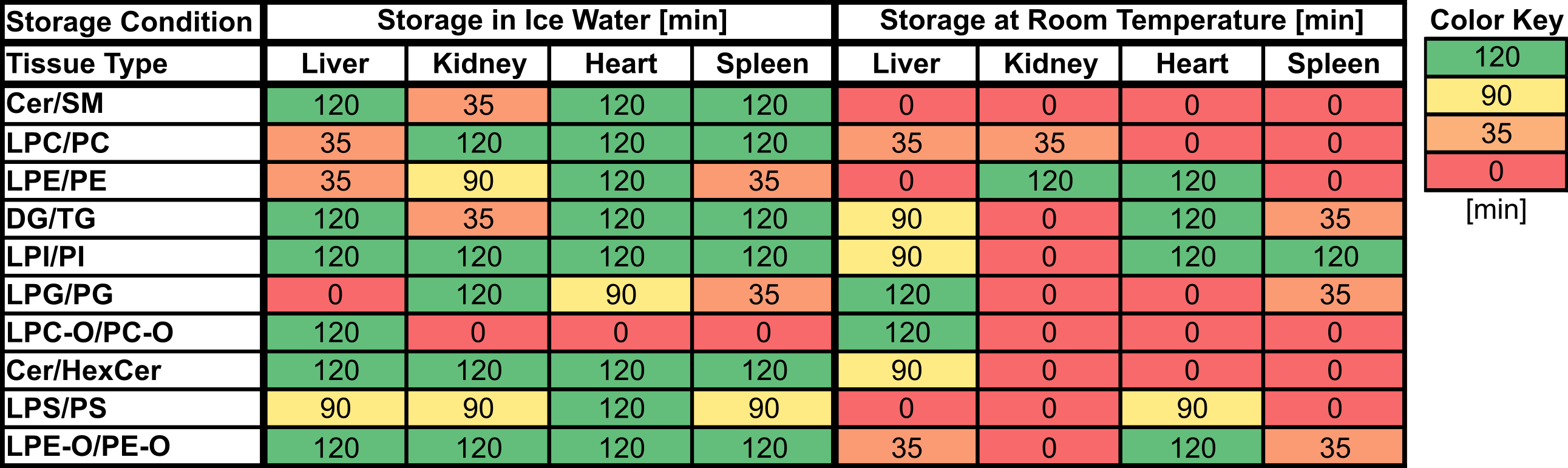

Supplement: Supplementary file 1 [file metabolites-13-00504-s001.zip › Figure S11_dpi300_2023-03-24_ED.png]

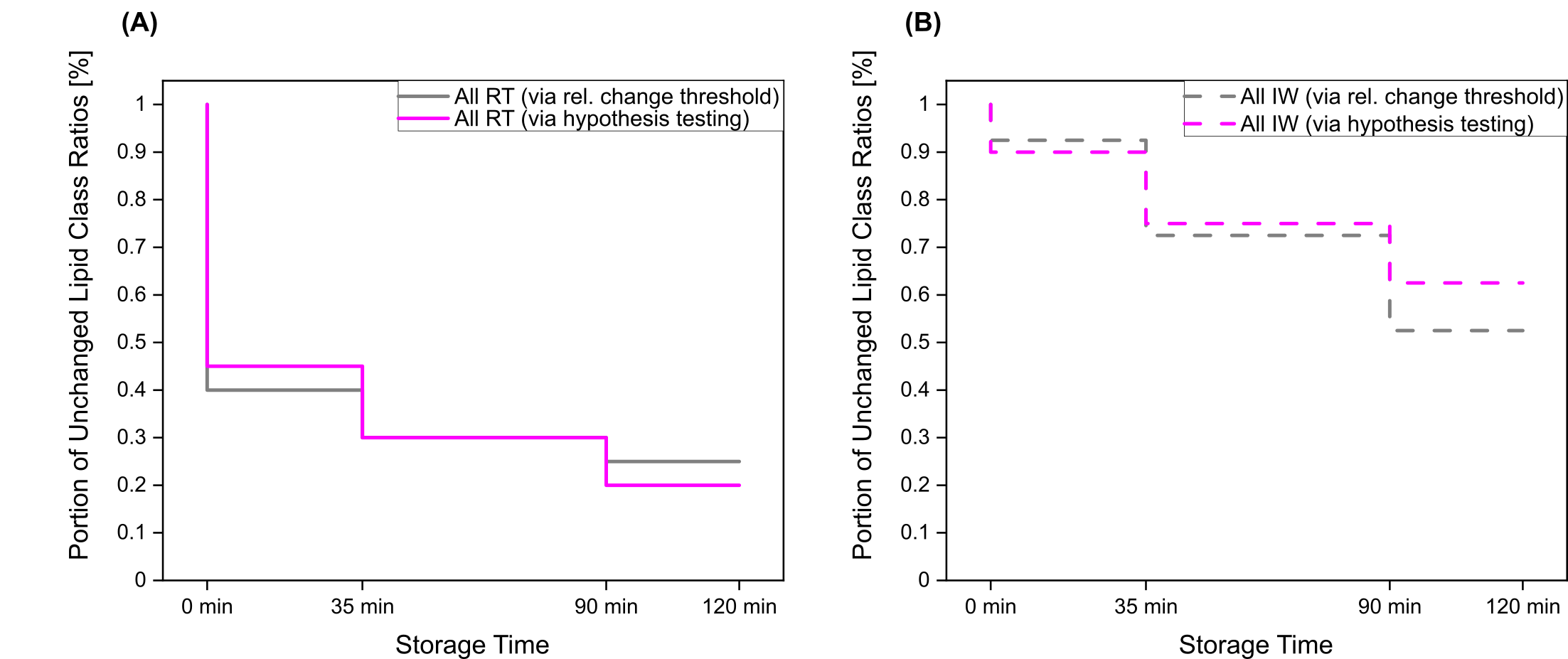

Supplement: Supplementary file 1 [file metabolites-13-00504-s001.zip › Figure S12_dpi300_2023-03-23_ED.png]

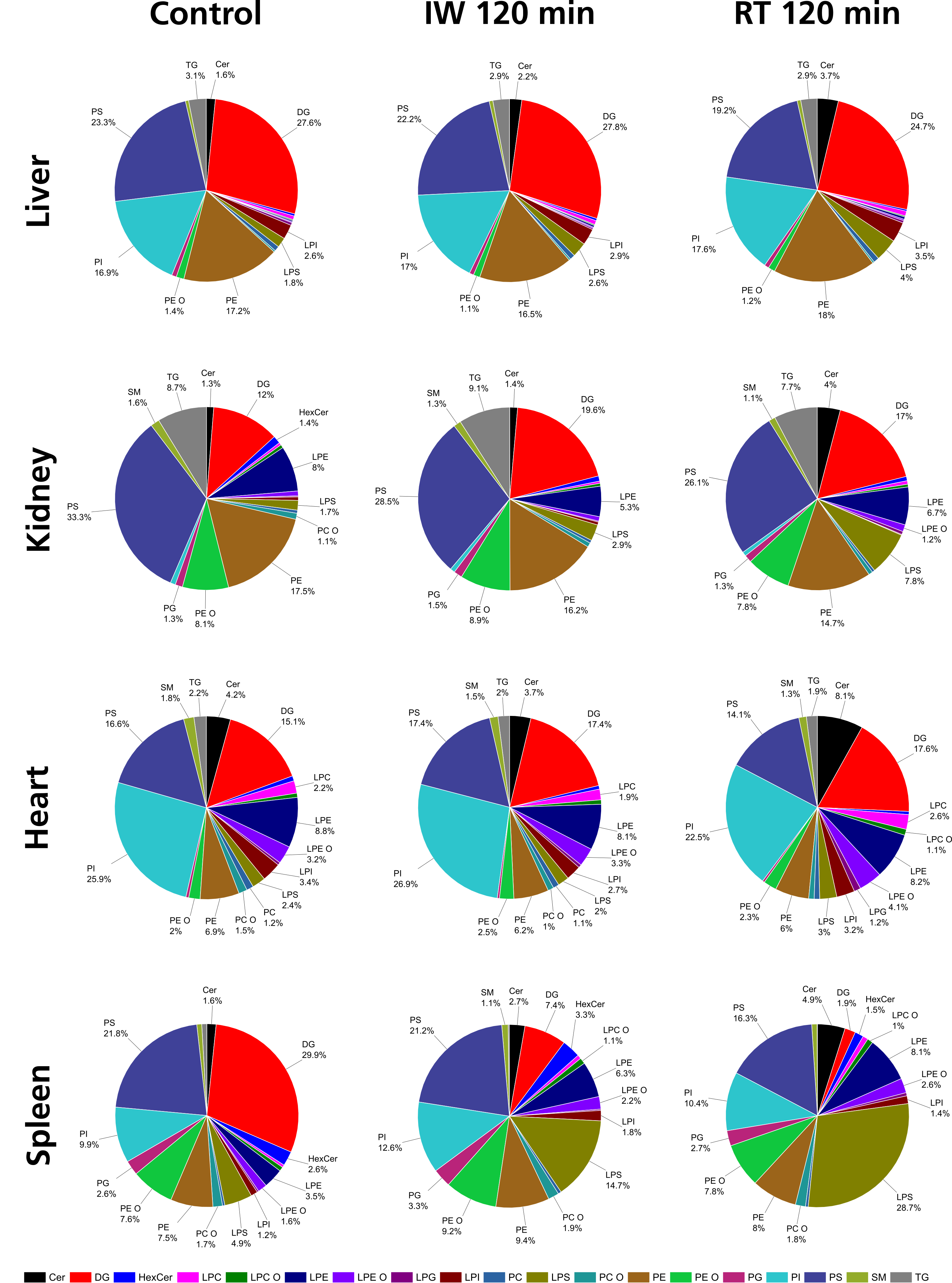

Supplement: Supplementary file 1 [file metabolites-13-00504-s001.zip › Figure S13_dpi300_2023-03-24_ED.png]

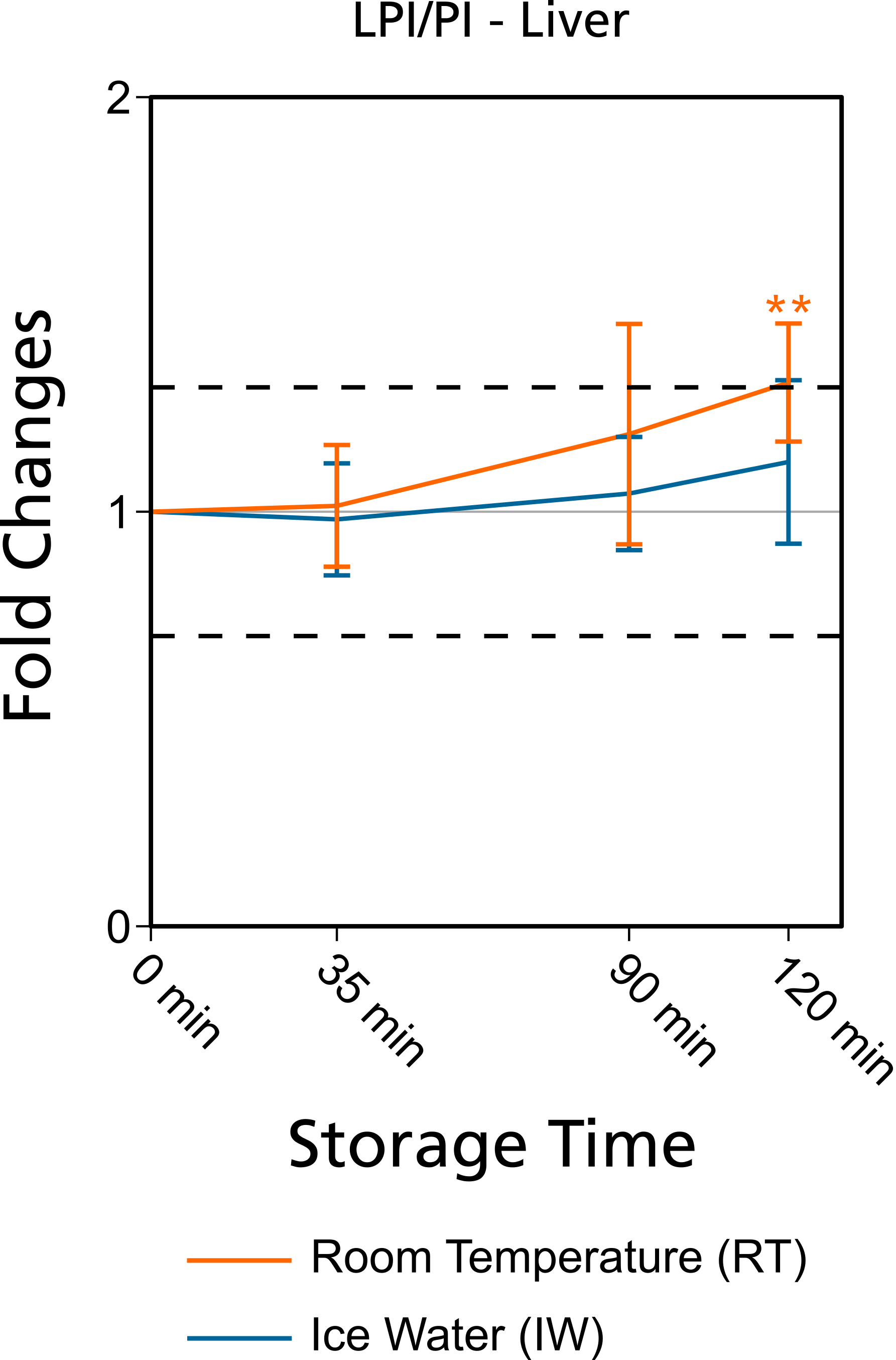

Supplement: Supplementary file 1 [file metabolites-13-00504-s001.zip › Figure S1_w Sign_dpi300_2023-03-22_ED.png]

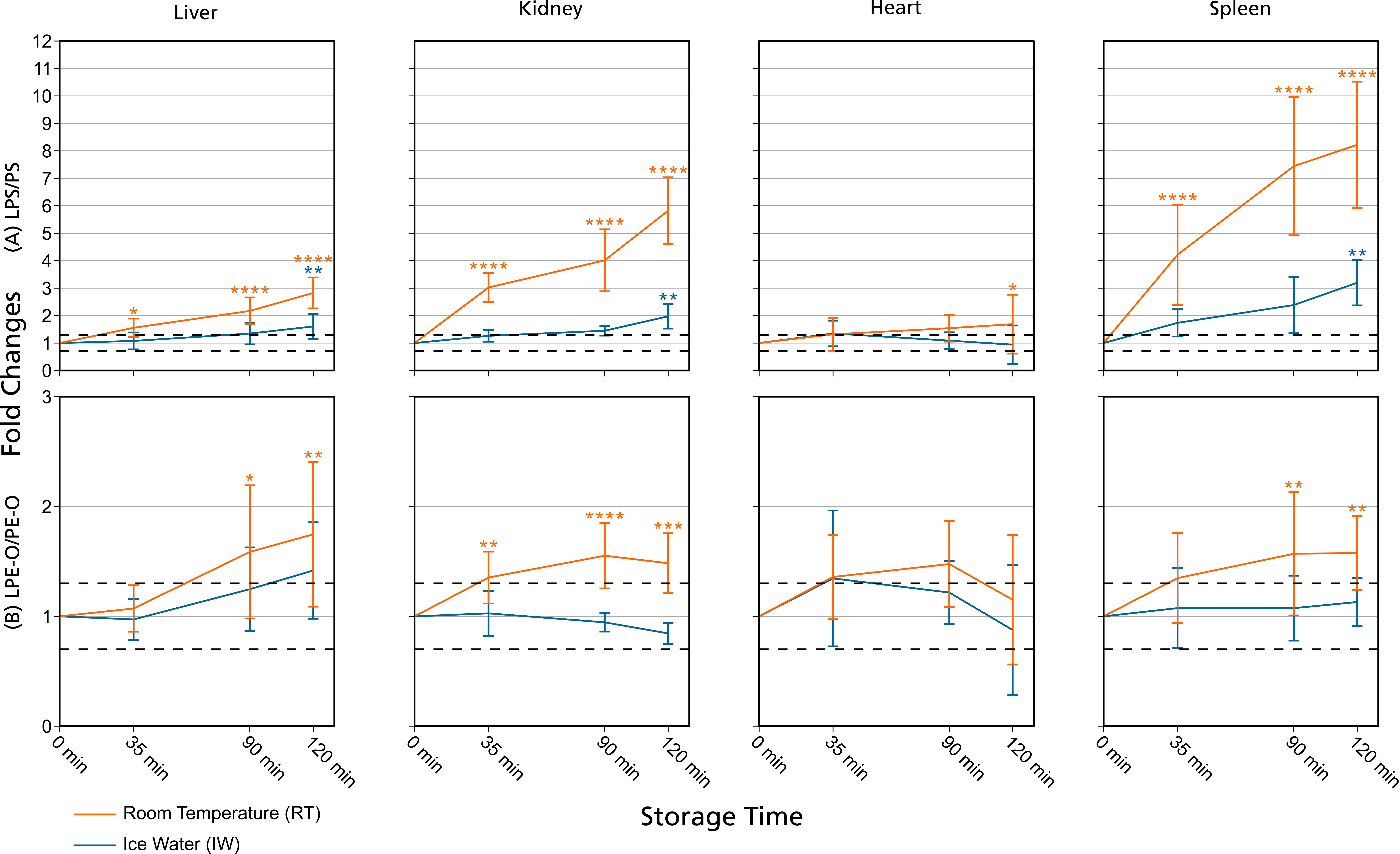

Supplement: Supplementary file 1 [file metabolites-13-00504-s001.zip › Figure S2_LPS-PS_LPEO-PEO_w Sign_dpi300_2023-03-22_ED.png]

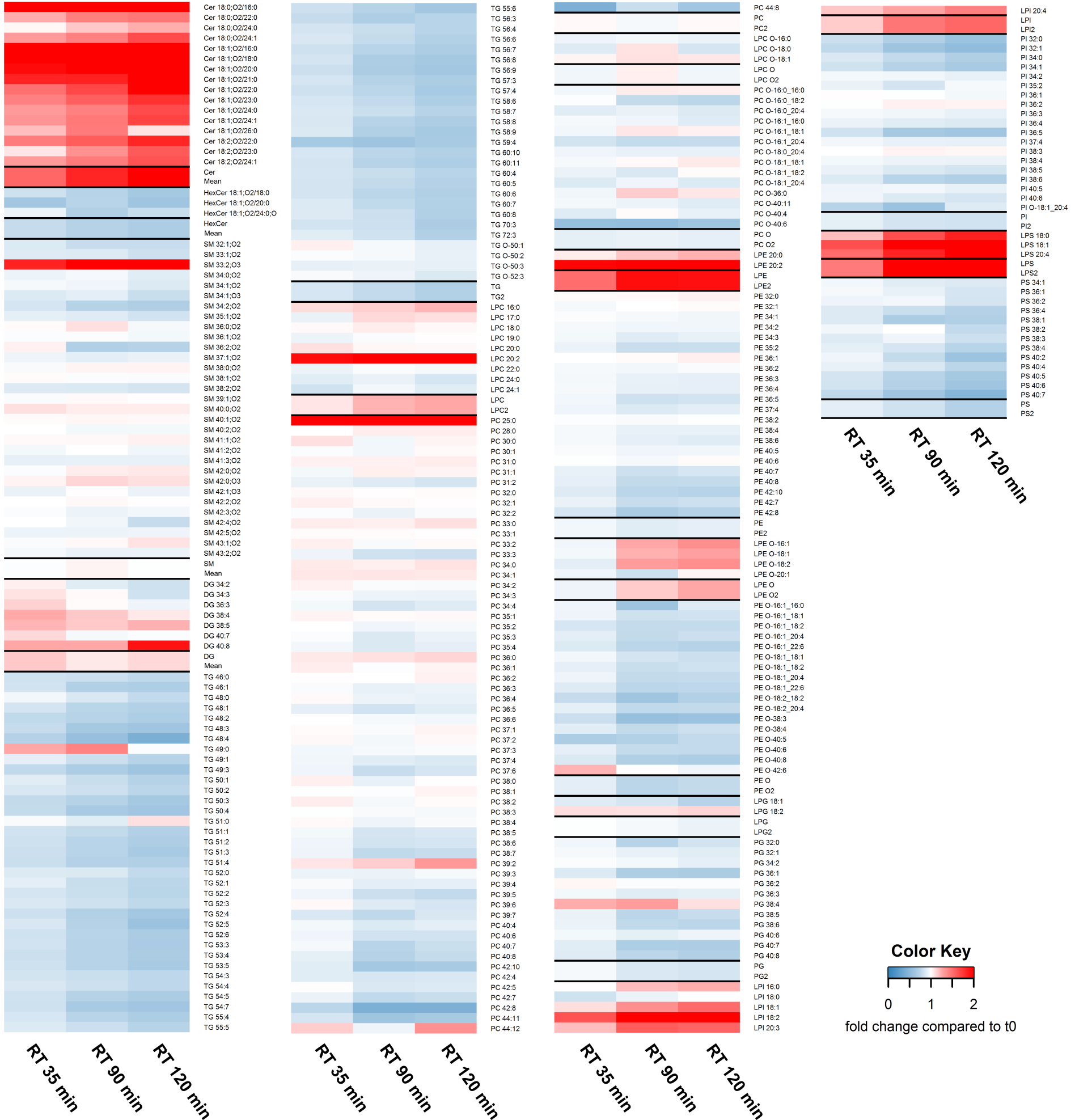

Supplement: Supplementary file 1 [file metabolites-13-00504-s001.zip › Figure S3_dpi300_2023-03-24_ED.png]

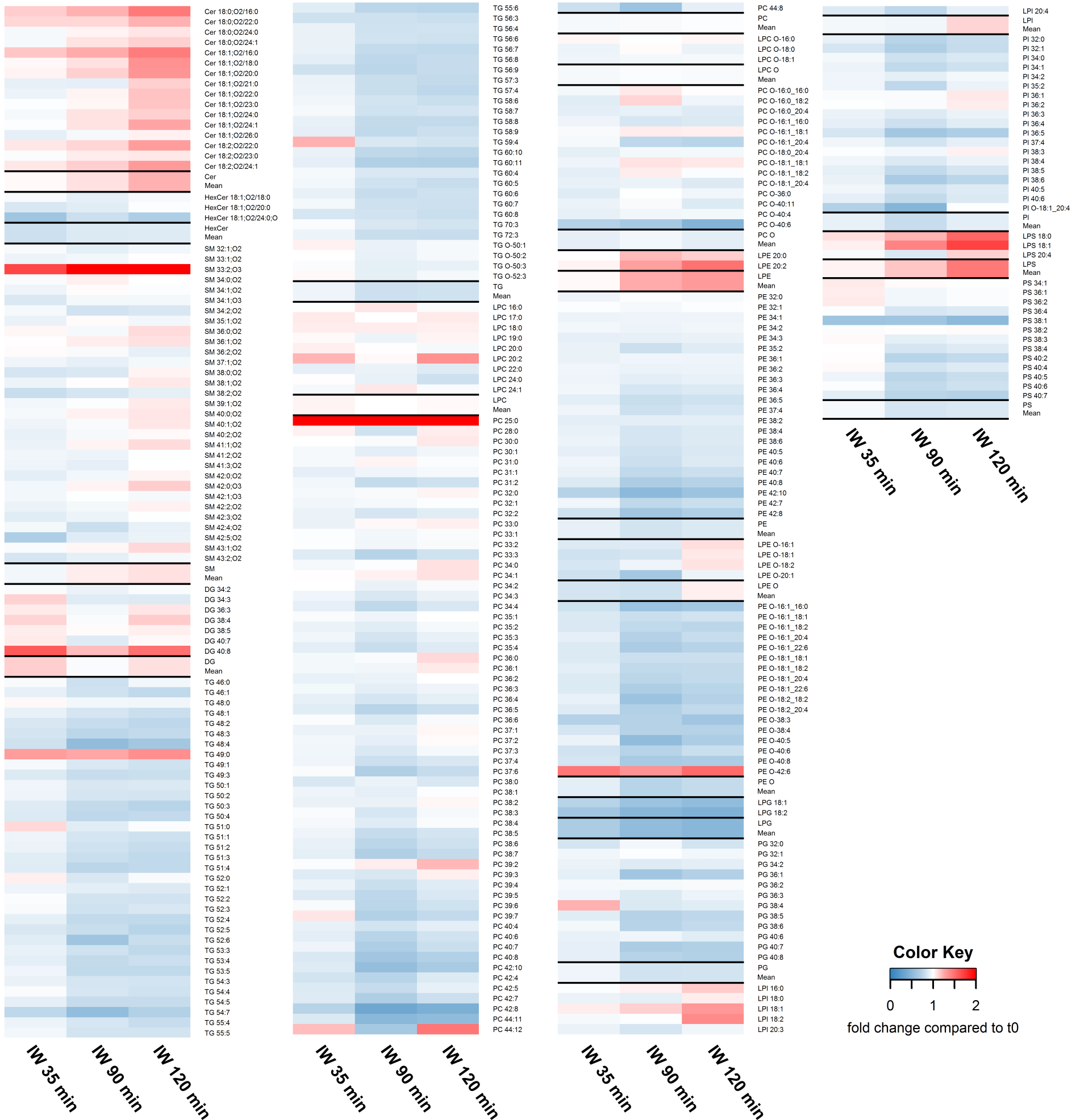

Supplement: Supplementary file 1 [file metabolites-13-00504-s001.zip › Figure S4_dpi300_2023-03-24_ED.png]

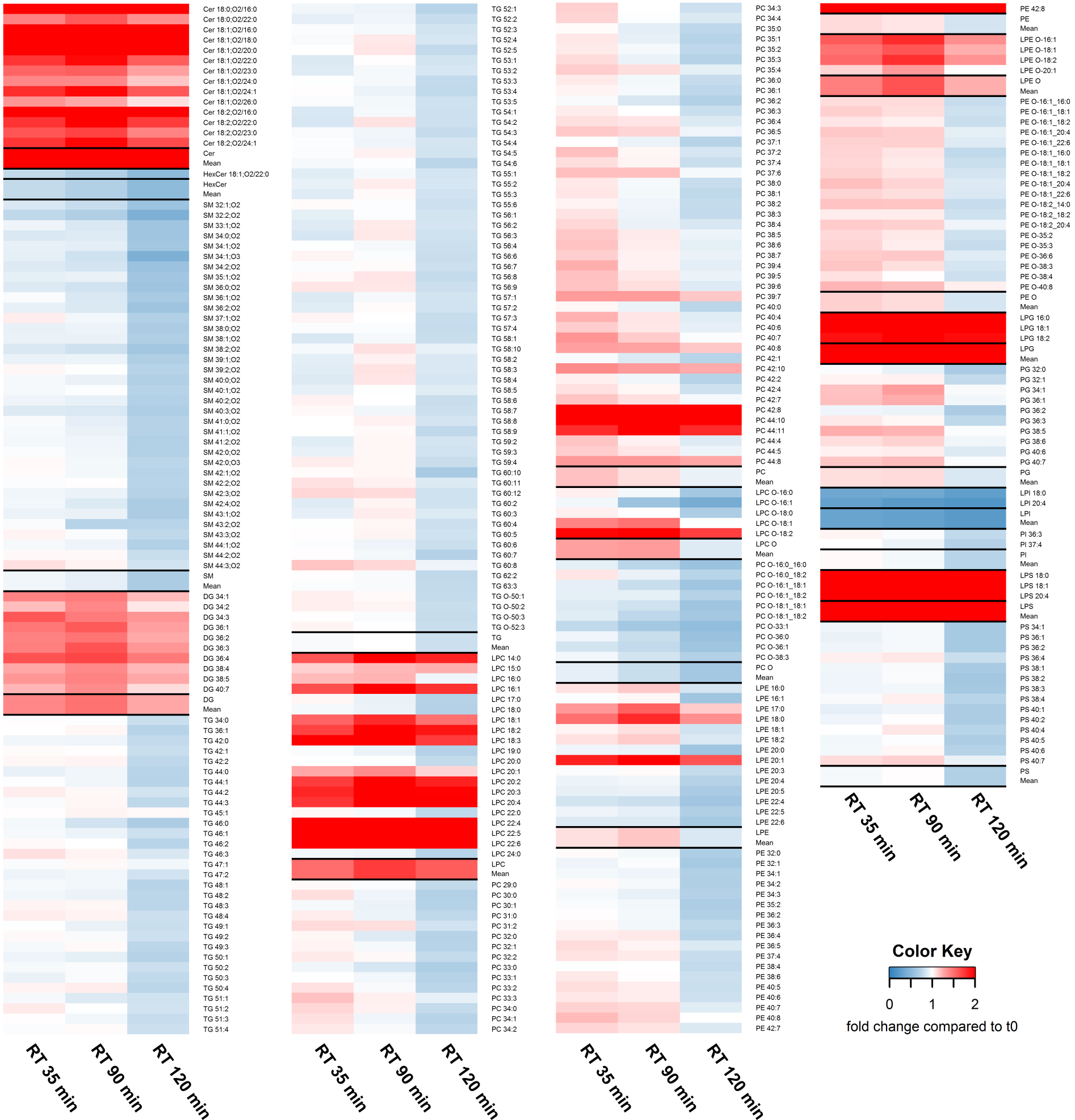

Supplement: Supplementary file 1 [file metabolites-13-00504-s001.zip › Figure S5_dpi300_2023-03-24_ED.png]

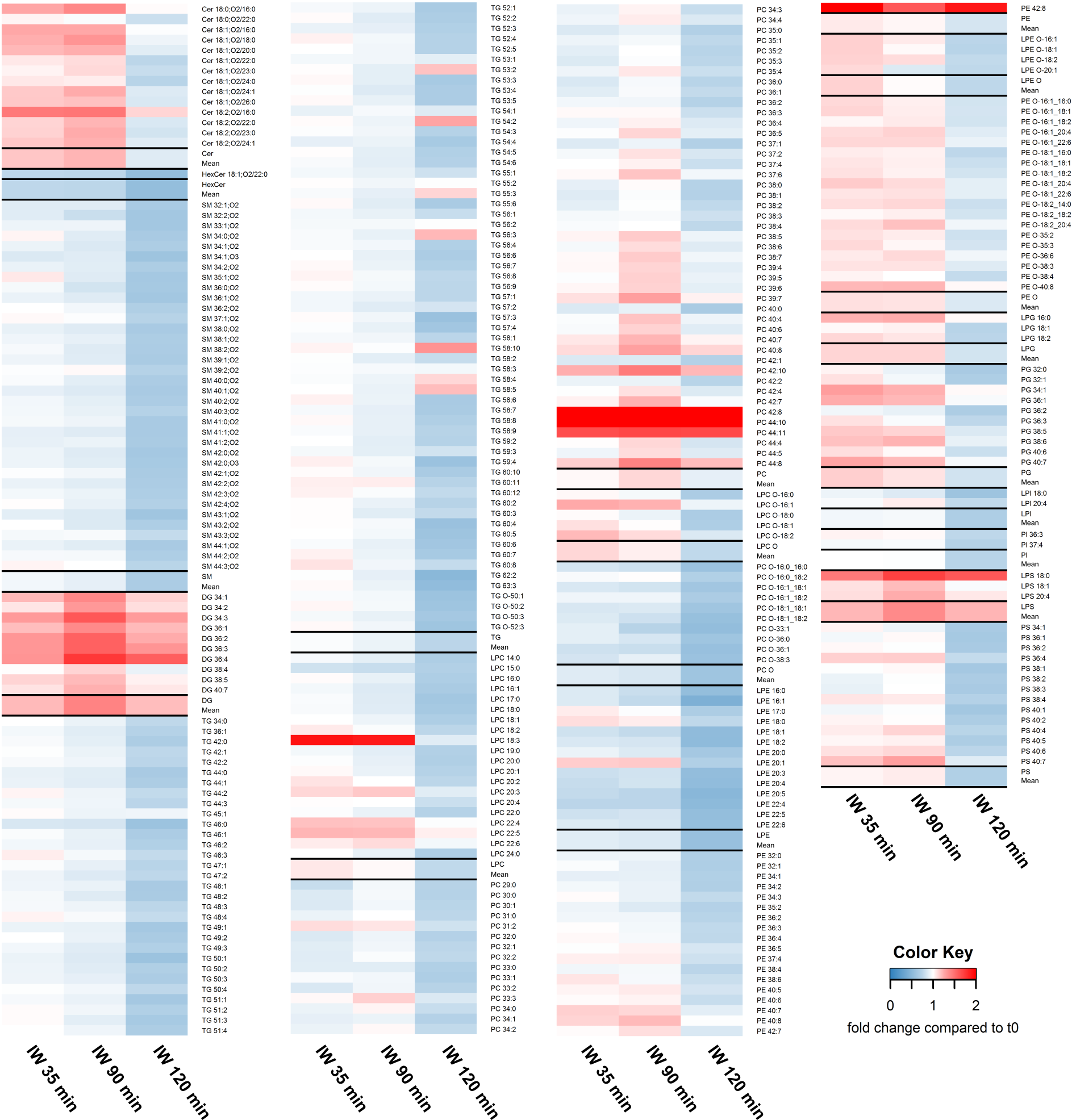

Supplement: Supplementary file 1 [file metabolites-13-00504-s001.zip › Figure S6_dpi300_2023-03-24_ED.png]

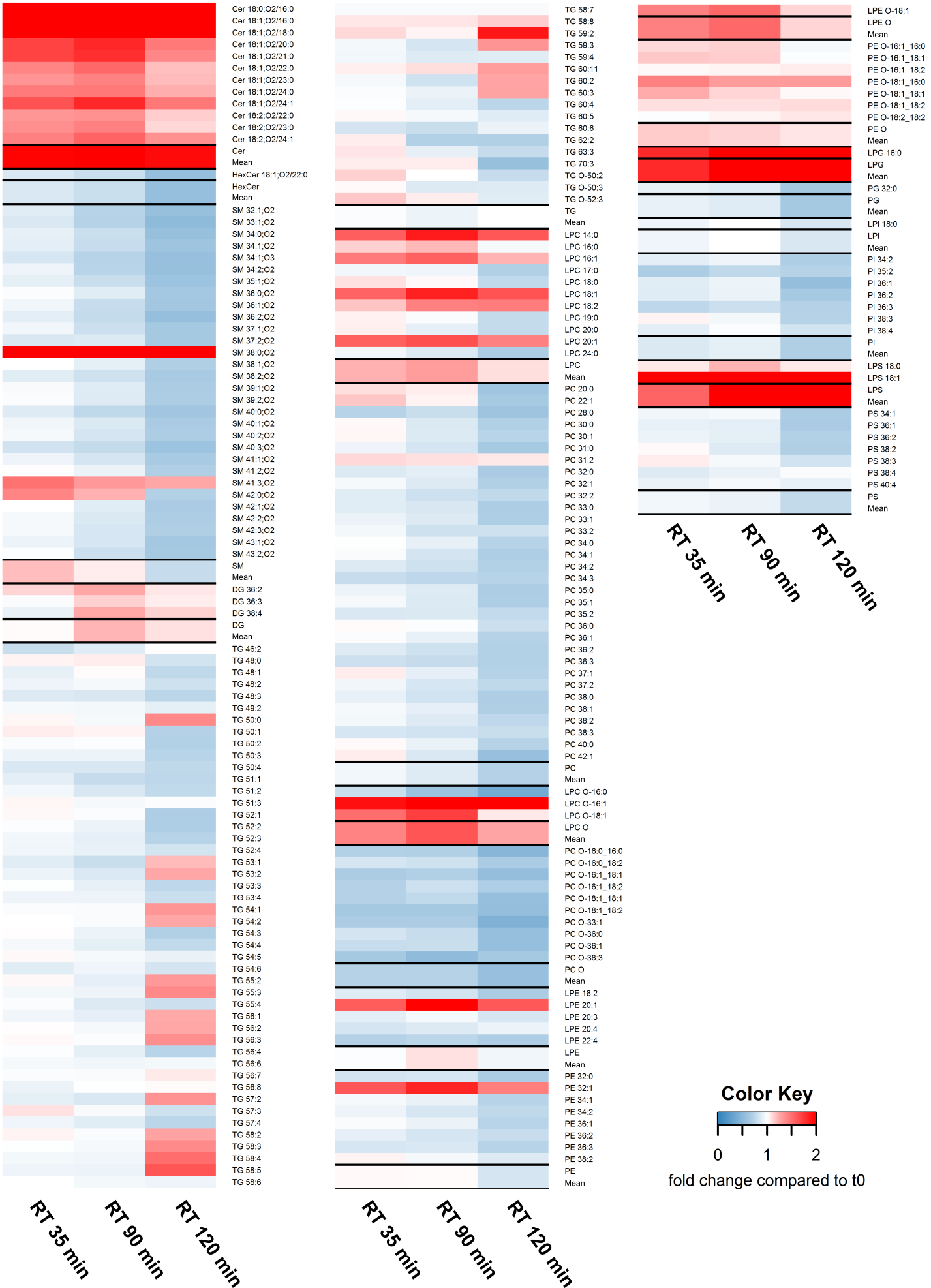

Supplement: Supplementary file 1 [file metabolites-13-00504-s001.zip › Figure S7_dpi300_2023-03-24_ED.png]

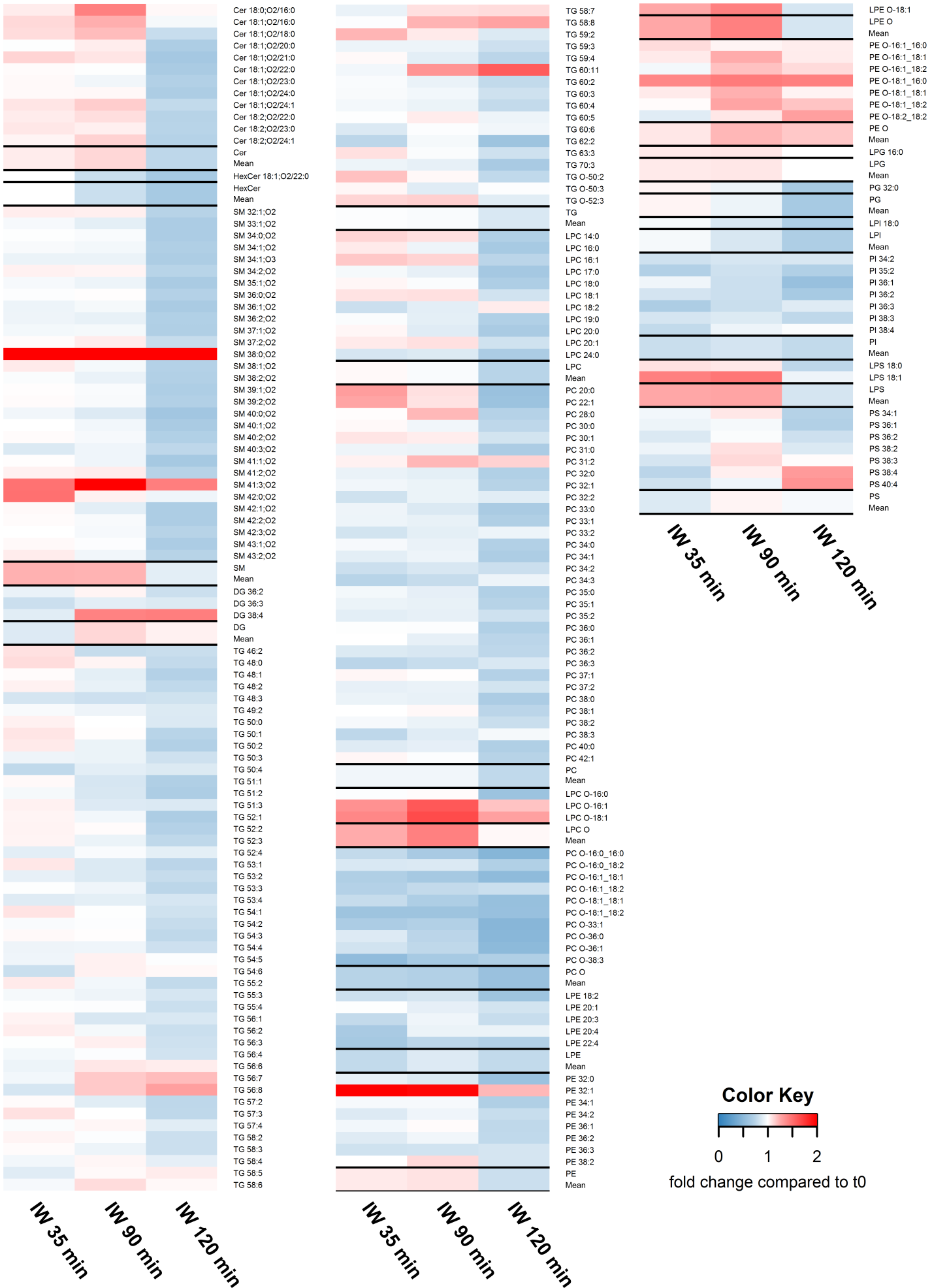

Supplement: Supplementary file 1 [file metabolites-13-00504-s001.zip › Figure S8_dpi300_2023-03-24_ED.png]

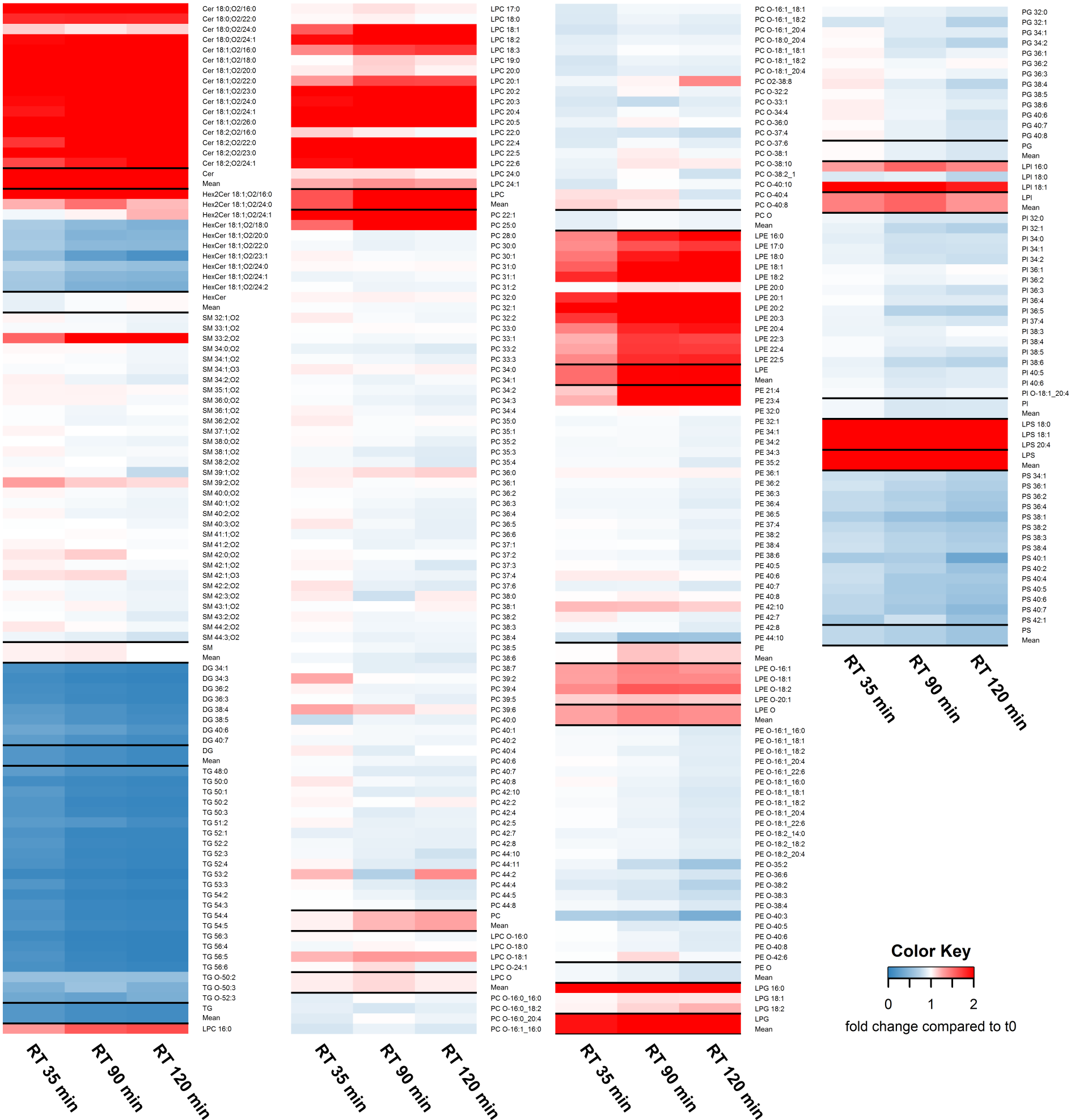

Supplement: Supplementary file 1 [file metabolites-13-00504-s001.zip › Figure S9_dpi300_2023-03-24_ED.png]
